# In-hospital survival and predictors of mortality among stroke patients at a tertiary hospital in Ghana: A retrospective cohort study

**DOI:** 10.1371/journal.pone.0340447

**Published:** 2026-01-07

**Authors:** Sulemana Baba Abdulai, Julius Kwabena Karikari, Penias Tembo, Theogene Habumugisha, William Tembo, Alhaji Ibrahim Cobbinah, Yeetey Enuameh

**Affiliations:** 1 Department of Epidemiology and Biostatistics, School of Public Health, Kwame Nkrumah University of Science and Technology, Kumasi, Ghana; 2 University Hospital, Kwame Nkrumah University of Science and Technology, Kumasi, Ghana; 3 Department of Epidemiology and Biostatistics, Arnold School of Public Health, University of South Carolina, Columbia South Carolina, United States of America; 4 Cancer Prevention and Control Program, University of South Carolina, Columbia South Carolina, United States of America; 5 Department of Global Public Health and Primary Care, Center for International Health, University of Bergen, Bergen, Norway; 6 Department of Internal Medicine, Neurology Division, University Teaching Hospital, Lusaka, Zambia; Independent Medical Researcher and Writer, UNITED KINGDOM OF GREAT BRITAIN AND NORTHERN IRELAND

## Abstract

**Introduction:**

Stroke is a leading cause of death and disability worldwide. In Ghana, national estimates show a prevalence of 7.9% and an incidence rate of 1.2%, placing a significant burden on the health system. This study aimed to estimate in-hospital survival rates and identify predictors of mortality among stroke patients admitted to Tamale Teaching Hospital (TTH).

**Methods:**

A retrospective electronic medical records review was conducted using data from the Lightwave Health Information Management System (LHIMS) and patients’ registry from January 1 2021 to December 31 2023. Kaplan-Meier survival curve was used to determine the survival rate of stroke patients (mean follow-up: 67 days). Cox proportional hazard regression determined the association between risk factors and survival time. Crude and adjusted hazard ratios with 95% confidence intervals were presented. A p-value of 0.05 was considered statistically significant.

**Results:**

A total of 998 stroke patients were included, of which 39.4% died. The overall survival rate was 21% at the end of follow-up (182 days). The survival probability for female stroke patients was lower compared to males. Female sex (AHR = 1.33, 95% CI: 1.07–1.65), Dagomba ethnicity (AHR = 1.31, 95% CI: 1.05–1.62), pneumonia (AHR = 1.59, 95% CI: 1.27–1.97), diabetes mellitus (AHR = 0.62, 95% CI: 0.47–0.82), systolic blood pressure ≥ 130 mmHg (AHR = 1.35, 95% CI: 1.06–1.73), and temperature ≥ 37.5°C (AHR = 1.79, 95% CI: 1.37–2.33) were predictors of mortality.

**Conclusion:**

Female stroke patients experienced higher mortality and lower survival compared to males. The identified mortality predictors underscore the importance of focused interventions to enhance survival outcomes at TTH. Given the retrospective design and possible unmeasured confounders, the lower mortality risk among diabetic patients should be interpreted with caution, and prospective studies are warranted to confirm these associations.

## Introduction

Stroke is an acute neurological dysfunction of the brain, spinal cord, or retina caused by focal ischemia (infarction) or haemorrhage, including cases due to cerebral venous thrombosis [1]. It is a multifaceted disease influenced by risk factors such as hypertension, diabetes, and heart failure [2]. Globally, stroke remains a leading cause of death and disability, responsible for significant health burdens, including an annual loss of approximately 1,484 disability-adjusted life years (DALYs) per 100,000 people [3]. Survivors often endure long-term physical, cognitive, and emotional challenges, imposing substantial demands on families, social, and healthcare systems [4].

In sub-Saharan Africa (SSA), the burden of stroke is rapidly increasing due to an epidemiological shift towards non-communicable diseases (NCDs) [5]. Stroke incidence and mortality rates in SSA are alarmingly high, with incidence reaching up to 1,460 cases per 100,000 people, and a three-year fatality rate exceeding 80% [6]. Challenges such as inadequate health infrastructure, limited neurologists, and low awareness of stroke symptoms exacerbate poor outcomes in the region [7]. In Ghana, stroke ranks among the top five causes of death, with concerning trends in rising admissions and a one-month hospital fatality rate of up to 43% [8,9]. Over the past three decades, the country has witnessed a consistent rise in stroke incidence, admissions, and mortality. For instance, stroke-related admissions in Kumasi increased from 5.32 per 1,000 in 1983 to 13.85 per 1,000 in 2010, with one-month fatality rates reaching 41% in the Central Region [9]. Key modifiable risk factors, including high salt intake, physical inactivity, and limited consumption of green leafy vegetables, are prevalent in Ghana [10]. Additionally, younger populations under 40 years are increasingly affected, posing significant socio-economic challenges [11].

The Tamale Teaching Hospital (TTH) in Northern Ghana, a major referral centre, reports high stroke-related mortality, with stroke ranking as the third leading cause of death in its Accident and Emergency Department [9]. Factors such as delayed presentation, socio-cultural barriers to healthcare, and resource constraints contribute to poor stroke outcomes [9]. While stroke-related mortality has been extensively studied, limited data exist on survival and its determinants, particularly in the Ghana context [7,12].

Despite significant advancements in stroke care, limited region-specific data on stroke survival and its determinants impede the development of targeted interventions in Ghana. Current research has predominantly focused on stroke mortality, with few studies on survival outcomes and their predictors [12–14]. This knowledge gap hinders the development of evidence-based stroke management strategies in LMICs, including the establishment of dedicated stroke units, the availability of diagnostic tools such as MRI machines, and the training of healthcare professionals essential for improving stroke care [15–17]. This study aims to address these gaps by investigating the in-hospital survival rate and predictors of mortality among stroke patients admitted to the Tamale Teaching Hospital, Ghana. The availability of evidence on the determinants of survival among stroke patients will help to inform healthcare planning, enhance resource allocation, and support the development of data-driven public health policies in Ghana. Furthermore, the study aligns with the WHO’s Agenda 2030 for Sustainable Development, particularly its goal of reducing mortality from major NCDs by one-third by 2030.

## Materials and methods

### Study design

This study employed a retrospective cohort design using routinely collected hospital data. Stroke patients admitted to Tamale Teaching Hospital between 2021 and 2023 were followed from admission until discharge or in-hospital death. The retrospective cohort design was appropriate for identifying predictors of in-hospital mortality among stroke patients using existing clinical records.

### Patient identification

**Fig 1:**
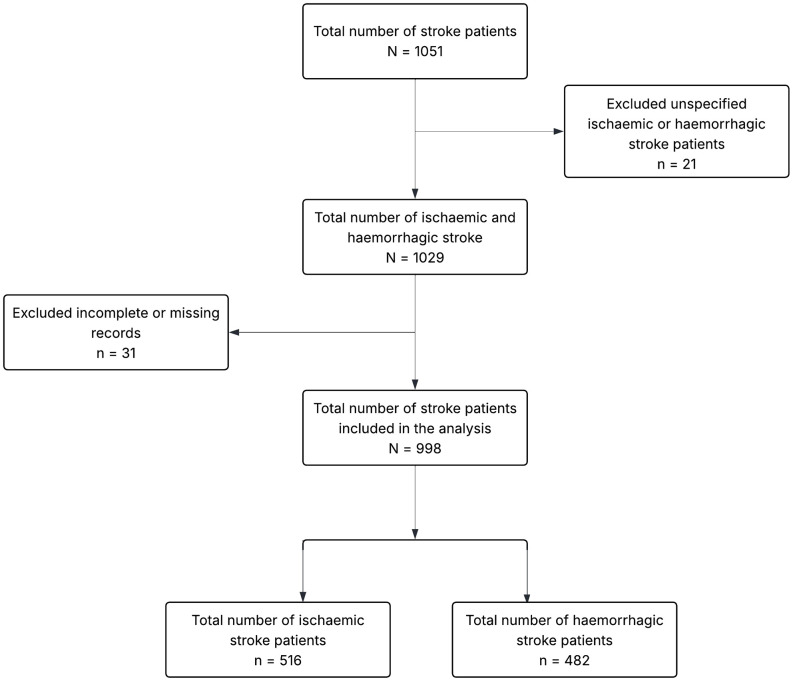
Identification and eligibility criteria used for the study population in the analysis.

We conducted a retrospective review of electronic medical records using the Lightwave Health Information Management System (LHIMS) to identify all patients admitted with a diagnosis of stroke at the Tamale Teaching Hospital (TTH) between January 1, 2021, and December 31, 2023. Medical records were accessed for research purposes between April 15 and June 15, 2024. The study included patients aged 18 years and above who were diagnosed with either ischemic or hemorrhagic stroke during the study period. No a priori sample size or power calculation was performed because the study included all eligible stroke cases within the 3 years. Patients were excluded if their medical records were incomplete or missing, or if the type of stroke was not specified as either ischemic or hemorrhagic (Fig 1). The authors had no access to information that could identify individual participants at any point during or after data collection. All data used for the study were fully anonymised prior to analysis to ensure the privacy and confidentiality of patients. The study adhered to the principles of the Declaration of Helsinki for research involving human participants. Ethical approval for the study was obtained from the Committee on Human Research, Publications and Ethics at the Kwame Nkrumah University of Science and Technology (KNUST), under approval number CHRPE/AP/237/24. Given the retrospective nature of the study, the requirement for informed consent was waived.

### Study setting

The study was centred at the Tamale Teaching Hospital (TTH) in Tamale, Northern region. The hospital is a referral centre for all other hospitals within the five regions, including the Northeast, Upper East, Savanna, and Upper West [9]. The hospital serves an approximate population of over 4 million people [18]. It is currently Ghana’s third-largest tertiary specialised healthcare facility [19]. In addition to providing medical care to patients, the hospital acts as a teaching facility for the University for Development Studies and many nursing colleges in the region [9]. At the time of the study, stroke care was provided within the general medical wards, as the hospital did not have a dedicated stroke unit. Stroke patients were managed by a multidisciplinary team comprising specialist physicians (including an endocrinologist and a gastroenterologist), medical officers, nurses, pharmacists, and physiotherapists. As a high-volume referral facility, TTH manages significant numbers of stroke patients; for example, 105 ischemic stroke admissions were recorded between January and October 2021 [9].

### Study variables

The event of interest was in-hospital death. Survival time was defined as the number of days from admission to death, with discharge alive treated as a censoring event. No post-discharge follow-up data were available. Predictors were grouped into demographic/baseline characteristics (age, sex, educational level, occupation, ethnicity, family history of stroke, smoking status, alcohol intake, body temperature, systolic and diastolic blood pressure, heart rate, respiratory rate, and Glasgow Coma Score) and clinical characteristics (family medical history, hypertension, diabetes mellitus, kidney disease, pneumonia, stroke type, atrial fibrillation, right and left upper limb weakness, seizures, aphasia, dysarthria, and dysphagia) (**Table 1**). Dates of admission and discharge or death were collected to compute time to in-hospital death or censoring.

**Table 1:** Demographic/baseline and clinical characteristics of stroke patients at TTH.

Characteristics	Frequency (N = 998)	Percentage (%)
**Age**		
< 45	178	17.84
45-65	441	44.19
≥ 66	379	37.98
**Sex**		
Male	546	54.71
Female	452	45.29
**Occupation**		
Unemployed	281	28.16
Employed	616	61.72
Retired	101	10.12
**Educational status**		
No education	705	70.64
Basic	40	4.01
SHS	105	10.52
Tertiary	148	14.83
**Ethnicity**		
Dagombas	521	52.20
Gonjas	35	3.51
Others^1^	442	44.29
**Family history of stroke**		
No	966	96.79
Yes	32	3.21
**Smoking**		
No	937	93.89
Yes	61	6.11
**Alcohol intake**		
No	882	88.38
Yes	116	11.62
**Temperature (°C)**		
< 37.5	859	86.07
≥ 37.5	139	13.93
**Systolic BP (mmHg)**		
< 130	311	31.16
≥ 130	687	68.84
**Diastolic BP (mmHg)**		
< 80	312	31.26
≥ 80	686	68.74
**Heart rate (bpm)**		
≤ 100	371	37.17
> 100	627	62.83
**Respiratory rate (breaths/min)**		
≤ 20	548	54.91
> 20	450	45.09
**GCS level**		
< 9	274	72.55
≥ 9	724	27.45
**Hypertension**		
No	214	21.44
Yes	784	78.56
**Diabetes**		
No	787	78.86
Yes	211	21.14
**Kidney disease**		
No	975	97.70
Yes	23	2.30
**Pneumonia**		
No	635	63.63
Yes	363	36.37
**Stroke type**		
Ischemic	516	51.70
Haemorrhagic	482	48.30
**Atrial fibrillation**		
No	995	99.70
Yes	3	0.30
**Right upper limb weakness**		
No	672	67.33
Yes	326	32.67
**Left upper limb weakness**		
No	710	71.14
Yes	288	28.86
**Right lower limb weakness**		
No	641	64.23
Yes	357	35.77
**Left lower limb weakness**		
No	674	67.54
Yes	324	32.46
**Seizures**		
No	869	87.07
Yes	129	12.93
**Aphasia**		
No	653	65.43
Yes	345	34.57
**Dysarthria**		
No	848	84.97
Yes	150	15.03
**Dysphagia**		
No	849	85.07
Yes	149	14.93
**Urea (mmol/L)**		
Median (IQR)	5.9 (4.82)	
**Creatinine(µmolL)**		
Median (IQR)	82.6 (60.30)	
**Bilirubin (µmolL)**		
Median (IQR)	11.5 (11.99)	
**Random blood sugar (mmol/L)**		
Median (IQR)	7.7(3.20)	

**1 = Wala, Kusasi, Frafra;**

^1^ **basic (1–9 years of formal education, primary and junior high school), SHS (Senior High School, 10–12 years of formal education), and Tertiary (≥13 years, post-secondary education).**

### Data sources and tools

Data for this study were extracted from the Lightwave Health Information Management System (LHIMS) and patient registers at the Tamale Teaching Hospital (TTH) for the years 2021–2023.

A structured checklist, developed in accordance with the RECORD (Reporting of studies Conducted using Observational Routinely collected Data) guidelines for studies using routinely collected health data, was used to extract information from LHIMS and the physical registers. The checklist ensured systematic and standardised retrieval of key variables, including sociodemographic characteristics, stroke type, comorbidities, and treatment outcomes for all patients admitted to the medical ward with a confirmed stroke diagnosis.

### Data analysis

Data extracted from LHIMS were entered into Excel, cleaned, and subsequently exported to STATA V.17 for analysis, an appropriate and robust platform for time-to-event analyses [20]. Univariable descriptive statistics, including frequencies and percentages, were presented in tables, with means and standard deviations for normally distributed continuous variables, and medians with interquartile ranges for skewed variables. All continuous variables were assessed for normality using the Shapiro–Wilk test, and their distributions were visualised using histograms. Kaplan–Meier survival curves were used to estimate time to in-hospital death, with discharge treated as a censoring event, and to describe overall survival probabilities. A log-rank test was performed to assess the difference between sex (male and female).

A stepwise Cox proportional hazards regression model was applied to identify variables associated with in-hospital mortality. Hazard ratios were used to measure the strength of these associations. The Schoenfeld residual test was conducted to verify the proportional hazards assumption, with variables meeting a p-value > 0.05 considered to satisfy the assumption. A bivariable Cox proportional hazards model was fitted for all predictors and variables with a p-value of less than 0.25 in order not to leave out important variables that may influence the outcome, and these were selected for inclusion in the stepwise multivariable Cox regression model. Multicollinearity among the predictors included in the multivariable model was assessed using the Variance Inflation Factor (VIF), and predictors with a VIF < 5 were included in the final model. [Mean VIF = 1.64, Max VIF = 2.63, Min VIF = 1.05], indicating no significant multicollinearity among the predictors. Statistical significance was set at p-value < 0.05 and a 95% confidence interval (CI).

### Patient and Public Involvement

Patients and members of the public were not involved in the design, conduct, reporting, or dissemination of this study, as it relied entirely on retrospectively collected hospital records.

## Results

### Baseline demographic and clinical characteristics of stroke patients

**Fig 2:**
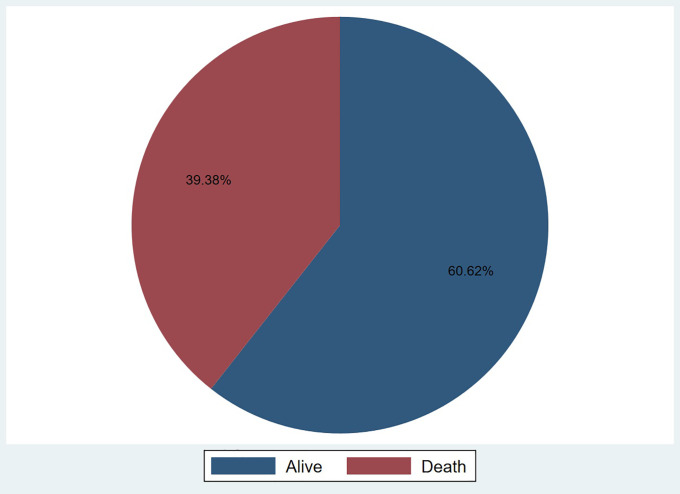
Admission outcome of stroke patients at TTH.

Most patients were aged between 45 and 65 years (44.19%). More than half of the patients were male (54.71%). Regarding occupation, most were employed (61.72%). Majority had no formal education (70.64%). Ethnically, Dagombas represented the largest group (52.20%). Most patients reported no family history of stroke (96.79%), were non-smokers (93.89%), and did not consume alcohol (88.38%). Clinically, most patients presented with a temperature below 37.5°C (86.07%) and had systolic and diastolic blood pressures of ≥130 mmHg (68.84%) and ≥80 mmHg (68.74%), respectively. A heart rate above 100 bpm was observed in 62.83% of patients, while 54.91% had a respiratory rate of 20 or fewer breaths per minute. The majority of patients had a Glasgow Coma Scale (GCS) score of ≥9 (72.55%). Hypertension was the most common, affecting 78.56% of patients, followed by diabetes (21.14%). Only a small proportion had kidney disease (2.30%). Pneumonia was documented in 36.37% of cases. Ischemic stroke was slightly more common than haemorrhagic stroke, accounting for 51.70% and 48.30% of cases, respectively. Atrial fibrillation was rare, reported in only 0.30% of patients. Regarding motor function and neurological deficits, the most common presentation was right upper limb weakness (32.67%), followed by left upper limb weakness (28.86%). Right lower limb weakness was observed in 35.77% of patients, while left lower limb weakness occurred in 32.46%. Seizures were noted in 12.93% of patients. Aphasia was present in 34.57% of cases, dysarthria in 15.03%, and dysphagia in 14.93% (**Table 1**). Approximately 60.62% of patients survived their admission, while 39.38% died during hospitalisation (**Fig 2**).

### Survival probability of stroke patients

**Fig 3:**
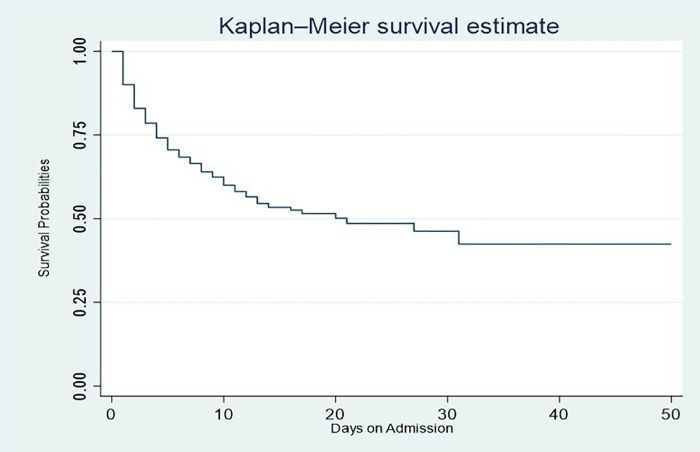
Overall Kaplan-Meier survival curve of stroke patients at TTH.

The overall survival probability was 21.0%, with a mean survival time of 67 days (95% CI: 42–91) (**Fig 3**). The results also show a decline in survival rates with longer hospital stays. The probability of survival was 90.0% on the 1st day, 71.0% on the 5th day, and 60.0% on the 10th day. By the end of the 50-day observation period, although some patients continued to survive, the survival probability had dropped significantly to 42.5%.

**Fig 4:**
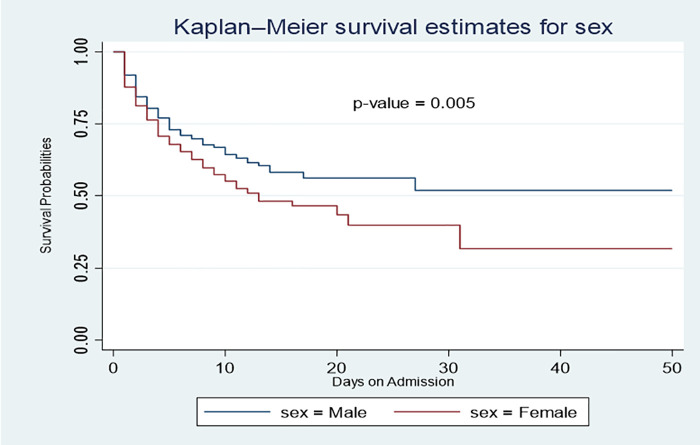
Kaplan-Meier survival analysis by stroke type.

Hemorrhagic stroke patients show shorter survival times than ischemic stroke patients, with a median survival of 31 days for ischemic stroke and 14 days for hemorrhagic stroke (**Fig 4**).

**Fig 5:**
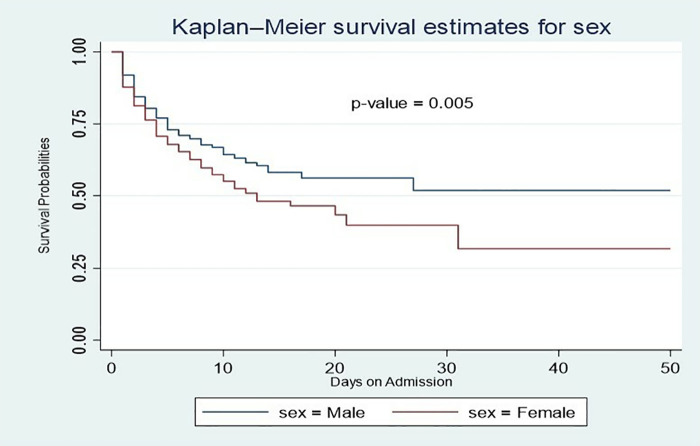
Kaplan-Meier survival analysis by sex.

Throughout the follow-up period, males consistently demonstrate a higher survival probability than females. The survival rate for females declined sharply, indicating a higher mortality rate over the same timeframe (**Fig 5**). For instance, on the 5th day, the survival probability for females was 68.0%, compared to 73.0% for males, and on the 10th day, it was 64.0% for males and 55.0% whereas for females. The median survival time for females was 13 days (95% CI: 10–31), while for males, it was 17 days.

### Sociodemographic and clinical predictors of in-hospital mortality among stroke patients

Patients aged 45–65 years had a significantly higher hazard of death compared to those younger than 45 years (AHR = 1.28; 95% CI: 1.03–1.58). Female patients had a significantly 33% higher risk of mortality compared to males (AHR = 1.33; 95% CI: 1.07–1.65). Educational status was also significantly associated with mortality. Patients with basic education had a 54% lower risk of death compared to those with no education. Among ethnic groups, Dagombas had a 31% increased risk of death compared to other groups (AHR = 1.31; 95% CI: 1.05–1.62). Patients with diabetes had a 39% lower risk of mortality compared to non-diabetics (AHR = 0.62; 95% CI: 0.47–0.82). Similarly, individuals presenting with right lower limb weakness had a 33% lower risk of death than those without the condition (AHR = 0.67; 95% CI: 0.53–0.85). Patients with elevated temperature (≥ 37.5°C) had a 79% higher hazard of death (AHR = 1.79; 95% CI: 1.37–2.33), while those with pneumonia had a 1.59 times higher risk of mortality than patients without pneumonia (AHR = 1.59; 95% CI: 1.27–1.97). Additionally, patients with systolic blood pressure ≥ 130 mmHg exhibited a 32% higher risk of death compared to those with lower systolic pressure (AHR = 1.35, 95% CI: 1.06–1.73). Interestingly, a heart rate above 100 bpm was associated with a significantly lower risk of mortality (AHR = 0.50, 95% CI: 0.40–0.62), as was a respiratory rate above 20 (AHR = 0.63, 95% CI: 0.49–0.79). These associations may reflect differences in stroke subtype or patient management and should be interpreted with caution. Higher oxygen saturation was significantly associated with better survival (AHR = 0.96, 95% CI: 0.95–0.98), while for each mmol/L increase in urea, the risk of mortality increased by 2% (AHR = 1.02; 95% CI: 1.01–1.03 per mmol/L) (Table 2).

**Table 2:** Sociodemographic and clinical predictors of in-hospital mortality among stroke patients.

Characteristics	Crude HR	95% CI	p-value	Adjusted HR	95% CI	p-value
**Age group**						
< 45	Ref			Ref		
45-65	1.25	0.92-1.70	0.154	1.28	1.03-1.58	0.027
≥ 66	1.07	0.78-1.48	0.663			
**Sex**						
Male	Ref			Ref		
Female	1.34	1.08-1.66	0.008	1.33	1.07-1.65	0.010
**Educational status**						
No education	Ref			Ref		
Basic	0.49	0.24-0.98	0.044	0.46	0.23-0.93	0.039
SHS	0.95	0.67-1.36	0.669			
Tertiary	0.57	0.40-0.83	0.003			
**Ethnicity**						
Others^1^	Ref			Ref		
Gonjas	0.62	0.29-1.33	0.219			
Dagombas	1.34	1.08-1.67	0.008	1.31	1.05-1.62	0.018
**Alcohol intake**						
No	Ref			Ref		
Yes	0.75	0.52-1.07	0.113	0.69	0.48-0.99	0.044
**Temperature (°C)**						
< 37.5	Ref			Ref		
≥ 37.5	2.26	1.75-2.91	<0.001	1.79	1.37-2.33	<0.001
**Systolic BP (mmHg)**						
< 130	Ref			Ref		
≥ 130	1.45	1.14-1.85	0.003	1.35	1.06-1.73	0.017
**Diastolic BP (mmHg)**						
< 80	Ref					
≥ 80	1.45	1.13 −1.85	0.003			
**Heart rate (bpm)**						
≤ 100	Ref			Ref		
> 100	0.40	0.32-0.49	<0.001	0.52	0.42-0.66	<0.001
**Respiratory rate (breaths/min)**						
< 20	Ref			Ref		
≥ 20	0.51	0.41-0.64	<0.001	0.60	0.47-0.76	<0.001
**GCS level**						
≥ 9	Ref					
< 9	1.23	0.97-1.53	0.094			
**Diabetes**						
No	Ref			Ref		
Yes	0.73	0.55-0.95	0.021	0.62	0.47-0.82	0.001
**Pneumonia**						
No	Ref			Ref		
Yes	1.89	1.53-2.34	<0.001	1.59	1.27-1.97	<0.001
**Stroke type**						
Ischaemic	Ref					
Haemorrhagic	1.17	0.95-1.45	0.144			
**Right upper limb weakness**						
No	Ref					
Yes	0.76	0.60-0.96	0.025			
**Right lower limb weakness**						
No	Ref					
Yes	0.72	0.57-0.91	0.005	0.67	0.53-0.85	0.001
**Dysarthria**						
No	Ref					
Yes	0.69	0.49-0.96	0.030			
**Urea (mmol/L)**						
	1.02	1.01-1.03	<0.001	1.02	1.01-1.03	<0.001
**Creatinine (µmolL)**						
	1.00	1.00-1.00	<0.001			
**Bilirubin (µmolL)**						
	1.01	0.99-1.02	0.196			
**Random blood sugar (mmol/L)**						
	1.01	1.00-1.02	0.022			

**HR = Hazard ratio, Ref = reference group, CI =Confidence interval, 1 = Wala, Kusasi, Frafra**

## Discussion

This study investigated the in-hospital survival rate and predictors of mortality among stroke patients admitted to the Tamale Teaching Hospital, Ghana. The study found that several factors, including female sex, Dagomba ethnicity, elevated temperature, pneumonia, higher systolic blood pressure, and increased urea levels, were associated with a higher risk of death due to stroke. Conversely, basic education, diabetes, right lower limb weakness, elevated heart and respiratory rates, and higher oxygen saturation were linked to improved survival outcomes.

The overall survival probability was high in the initial days of admission but decreased as follow-up time increased. Revealing further that a high number of patients died in the first 10 days after being diagnosed with stroke. This is comparable to a study in North West Ethiopia, with similar observations [4]. Also, the median survival time of 21 days was lower than in a study in Ethiopia that had a median survival time of 41 days [21]. This difference is likely explained by contextual factors such as variation in stroke severity and complication rates, health systems, and variation in sample size [4,21].

Males consistently showed a higher survival probability throughout the follow-up period, indicating a gender disparity in stroke outcomes. This aligns with findings from a study by [22] conducted in South Korea. The steep decline in survival rates among females, indicates a higher mortality rate, evident as early as the 5th-day post-stroke, where the survival probability for females was 68.0% compared to 73.0% for males. This pattern persists, with a significant gap by the 10th day, as survival probabilities drop to 55.0% for females and 64.0% for males. The median survival time further underscores this difference, with females showing a notably shorter median survival time of 13 days compared to 17 days for males. This disparity may be attributed to more severe post-stroke complications or other factors impacting female survival rates over time [23].

The findings also revealed that the odds of mortality were high among females compared to males. This aligns with a study in Ghana at the Korlebu Teaching Hospital [24]. Several factors may contribute to the higher burden of stroke-related mortality and disability among females. Sociocultural gender roles and biological differences influence stroke risk, assessment, treatment, and outcomes. The relationship between general stroke risk factors and female-specific risk variables differs considerably. Additionally, there are differences in how women experience stroke symptoms, respond to treatment, and recover after a stroke compared to men [23].

The study also revealed that stroke patients with no formal education faced a higher risk of mortality compared to those with education. Specifically, having a basic education was associated with approximately a 54% lower risk of death. This finding is consistent with studies conducted in Ghana and China [14,25]. This finding may be attributed to individuals with no education often displaying less healthy lifestyle behaviours and greater clinical risk factors for stroke, a pattern observed in both genders [26].

Also, the study found that individuals of Dagomba ethnicity had a higher risk of mortality following stroke. While direct comparisons are limited, this observation aligns with findings from the United States, where studies have consistently reported higher stroke mortality rates among Black populations [27,28]. A possible explanation is that cultural beliefs within certain ethnic groups may encourage reliance on traditional healing practices, leading to delays in seeking hospital care [29,30]. These disparities include a lack of awareness about stroke symptoms, delays in seeking timely treatment, and limited understanding of risk factors. Differences in attitudes, beliefs, and adherence to medical advice also vary across races and ethnicities.

The study revealed that pneumonia was also a predictor of mortality. The risk of mortality was high among stroke patients with pneumonia complications. This is consistent with a study in Ghana, Nigeria and Ethiopia [14,21,31]. The increased risk of mortality can be linked to several factors. Post-stroke patients frequently encounter swallowing difficulties and limited mobility, both of which can contribute to the development of lung infections like pneumonia [32]. Moreover, strokes can induce an immune response that heightens vulnerability to infections, disrupts the tracheal epithelium, diminishes lung clearance, and hampers the expulsion of secretions, further elevating the likelihood of pneumonia [33].

Interestingly, the study revealed that stroke patients with diabetes had a lower risk of mortality, which contrasts with findings observed in Ghana [14,34]. This finding needs to be further investigated as it is very counterintuitive unless something special is being done in the population or the health facility within which these cases are being picked

Furthermore, we found that patients presenting with right lower limb weakness had a lower risk of death compared to those without such weakness. Although this specific finding has limited direct support in existing literature, a study conducted in Canada reported that outcomes tend to be more favourable in individuals with motor strokes, particularly those presenting with monoparesis, defined as weakness in a single limb [35]. While this does not directly align with our findings, it suggests that isolated motor deficits may be associated with less severe stroke presentations, potentially contributing to improved survival. Overall, patients with motor stroke symptoms often experience fewer complications and shorter hospital stays, which may partly explain the observed association in our study. Also, these patients are more likely to experience symptom resolution by the time of hospital discharge, which further supports the observation that isolated motor deficits may predict better prognoses compared to more extensive motor involvement.

### Implications for practice and research

Despite the limitations, the findings of this study provide an important contribution with relevant implications for improving the survival of stroke patients. The identification of both demographic and clinical predictors of stroke mortality has important implications for clinical practice. High-risk patients, such as females, individuals with low or no education, those presenting with aphasia, pneumonia, elevated temperature, or high systolic blood pressure, may benefit from closer monitoring and aggressive management during the acute phase of stroke. The protective associations observed with diabetes suggest potential responsiveness to treatment that warrants further investigation. Early identification and prompt intervention for modifiable risk factors like diabetes could improve survival outcomes, especially during the critical first 10 days post-admission when mortality risk is highest.

## Strengths and limitations

This study provides valuable insights into the factors that influence survival among stroke patients in a real-world clinical setting. By using a multivariable Cox regression model, we were able to control for several demographic and clinical variables, helping us identify the key predictors of mortality more accurately. The study also benefits from a relatively large sample size and a three-year follow-up period, which strengthens the reliability of our findings. Importantly, by examining both social and clinical factors, we offer a more holistic picture of the challenges stroke patients face in our context.

That said, our study has a few limitations worth noting. Because it is based on retrospective data, we were limited to the information recorded in patients’ medical files, which were sometimes incomplete or missing key details. We also could not assess stroke severity scores (e.g., NIHSS, mRS) or functional outcomes at admission, which might have further clarified the survival patterns we observed. Although Kaplan–Meier survival analysis was used to compare survival between ischaemic and haemorrhagic stroke patients, this study did not conduct subtype-specific multivariable regression analyses to identify predictors of mortality within each stroke subtype. Also, detailed information on arterial territories in ischaemic stroke and cerebral localization of haemorrhagic stroke was not available. These limitations may have reduced the ability to detect subtype-specific prognostic factors. In addition, we only looked at what happened during the hospital stay, so we might have missed deaths that occurred after discharge.

## Conclusion

The study revealed that stroke patients treated at the Tamale Teaching Hospital face a declining probability of survival over time, with the highest risk of death occurring within the first 10 days of admission. To reduce preventable deaths, health system actions are needed including: (1) establishing or strengthening dedicated stroke care pathways or a stroke unit at TTH to ensure rapid assessment and standardized management; (2) prioritizing early monitoring and control of modifiable, high-risk comorbidities such as fever, pneumonia, and elevated systolic blood pressure; (3) implementing targeted screening and public-health outreach for high-risk groups and tailored education for patients and caregivers; and (4) ensuring protocols for infection prevention and early rehabilitation and discharge planning to reduce complications and length of stay. However, given the retrospective design and potential for residual confounding, large and prospective studies are still needed.

## Supporting information

S1 File Data used for the analysis. (XLSX)

## References

[1] Sacco RL, Kasner SE, Broderick JP, Caplan LR, Connors JJB, Culebras A, et al. An updated definition of stroke for the 21st century: a statement for healthcare professionals from the American Heart Association/American Stroke Association. Stroke. 2013;44(7):2064–89. doi: 10.1161/STR.0b013e318296aeca 23652265 PMC11078537

[2] Russell JBW, Charles E, Conteh V, Lisk DR. Risk factors, clinical outcomes and predictors of stroke mortality in Sierra Leoneans: A retrospective hospital cohort study. Ann Med Surg (Lond). 2020;60:293–300. doi: 10.1016/j.amsu.2020.10.060 33204420 PMC7649580

[3] GBD 2019 Stroke Collaborators. Global, regional, and national burden of stroke and its risk factors, 1990-2019: a systematic analysis for the Global Burden of Disease Study 2019. Lancet Neurol. 2021;20(10):795–820. doi: 10.1016/S1474-4422(21)00252-0 34487721 PMC8443449

[4] Walelgn N, Abyu GY, Seyoum Y, Habtegiorgis SD, Birhanu MY. The Survival Status and Predictors of Mortality Among Stroke Patients at North West Ethiopia. Risk Manag Healthc Policy. 2021;14:2983–94. doi: 10.2147/RMHP.S322001 34285612 PMC8286726

[5] Ayehu GW, Yitbarek GY, Jemere T, Chanie ES, Feleke DG, Abebaw S, et al. Case fatality rate and its determinants among admitted stroke patients in public referral hospitals, Northwest, Ethiopia: A prospective cohort study. PLoS One. 2022;17(9):e0273947. doi: 10.1371/journal.pone.0273947 36108071 PMC9477361

[6] Akinyemi RO, Ovbiagele B, Adeniji OA, Sarfo FS, Abd-Allah F, Adoukonou T, et al. Stroke in Africa: profile, progress, prospects and priorities. Nat Rev Neurol. 2021;17(10):634–56. doi: 10.1038/s41582-021-00542-4 34526674 PMC8441961

[7] Sarfo FS, Akpa OM, Ovbiagele B, Akpalu A, Wahab K, Obiako R, et al. Patient-level and system-level determinants of stroke fatality across 16 large hospitals in Ghana and Nigeria: a prospective cohort study. Lancet Glob Health. 2023;11(4):e575–85. doi: 10.1016/S2214-109X(23)00038-4 36805867 PMC10080070

[8] Edzie EKM, Gorleku PN, Dzefi-Tettey K, Idun EA, Amankwa AT, Aidoo E, et al. Incidence rate and age of onset of first stroke from CT scan examinations in Cape Coast metropolis. Heliyon. 2021;7(2):e06214. doi: 10.1016/j.heliyon.2021.e06214 PMC7892921 33659742

[9] Kumi F, Bugri AA, Adjei S, Duorinaa E, Aidoo M. Quality of acute ischemic stroke care at a tertiary Hospital in Ghana. BMC Neurol. 2022;22(1):28. doi: 10.1186/s12883-021-02542-9 35039001 PMC8762857

[10] Sanuade OA, Dodoo FN-A, Koram K, de-Graft Aikins A. Prevalence and correlates of stroke among older adults in Ghana: Evidence from the Study on Global AGEing and adult health (SAGE). PLoS One. 2019;14(3):e0212623. doi: 10.1371/journal.pone.0212623 30865654 PMC6415815

[11] Tawiah A. Stroke cases rising among young people. https://www.graphic.com.gh/news/general-news/stroke-cases-rising-among-young-people.html. 2023.

[12] Sarfo FS, Ovbiagele B. Key determinants of long-term post-stroke mortality in Ghana. J Neurol Sci. 2022;434:120123. doi: 10.1016/j.jns.2021.120123 34974202 PMC8979649

[13] Sarfo FS, Akassi J, Kyem G, Adamu S, Awuah D, Kantanka O-S, et al. Long-Term Outcomes of Stroke in a Ghanaian Outpatient Clinic. J Stroke Cerebrovasc Dis. 2018;27(4):1090–9. doi: 10.1016/j.jstrokecerebrovasdis.2017.11.017 29275059 PMC5845472

[14] Sarfo FS, Akassi J, Ofori E, Ovbiagele B. Long-term determinants of death after stroke in Ghana: Analysis by stroke types & subtypes. J Stroke Cerebrovasc Dis. 2022;31(9):106639. doi: 10.1016/j.jstrokecerebrovasdis.2022.106639 35926405 PMC9742008

[15] Baatiema L. The knowledge-practice gap: Evidence-based practice for acute stroke care in Ghana. 2018.

[16] Baatiema L, de-Graft Aikins A, Sarfo FS, Abimbola S, Ganle JK, Somerset S. Improving the quality of care for people who had a stroke in a low-/middle-income country: A qualitative analysis of health-care professionals’ perspectives. Health Expect. 2020;23(2):450–60. doi: 10.1111/hex.13027 31967387 PMC7104640

[17] Itanyi UD, Nnodu OE. Prevention of stroke and cognitive decline in pediatric population in resource-limited settings. Front Stroke. 2024;3. doi: 10.3389/fstro.2024.1390220 PMC12802630 41542266

[18] Nukpezah RN, Atanuriba GA. “It is an emotional rollercoaster!!!” Experiences of mothers of preterm newborns seeking care at a tertiary hospital in Ghana: a qualitative phenomenological study. BMJ Open. 2025;15(1):e093173. doi: 10.1136/bmjopen-2024-093173 39819941 PMC11751851

[19] Tamale Teaching Hospital. https://en.wikipedia.org/w/index.php?title=Tamale_Teaching_Hospital&oldid=1321995482 2025 2025 December 8.

[20] Cleves MA. An introduction to survival analysis using Stata. 3rd ed. College Station, Tex: Stata Press. 2010.

[21] Sahle Adeba T, Mekonen H, Alemu T, Alate T, Melis T. Survival status and predictor of mortality among adult stroke patients in Saint Paul’s hospital millennium medical college, Addis Ababa, Ethiopia. SAGE Open Med. 2022;10:20503121221112483. doi: 10.1177/20503121221112483 35924142 PMC9340903

[22] Kim J-S, Lee K-B, Roh H, Ahn M-Y, Hwang H-W. Gender differences in the functional recovery after acute stroke. J Clin Neurol. 2010;6(4):183–8. doi: 10.3988/jcn.2010.6.4.183 21264198 PMC3024522

[23] Kelly-Hayes M. Influence of age and health behaviors on stroke risk: lessons from longitudinal studies. J Am Geriatr Soc. 2010;58 Suppl 2(Suppl 2):S325-8. doi: 10.1111/j.1532-5415.2010.02915.x 21029062 PMC3006180

[24] Wiredu EK, Nyame PK. Stroke-related mortality at Korle Bu Teaching Hospital, Accra, Ghana. East Afr Med J. 2001;78(4):180–4. doi: 10.4314/eamj.v78i4.9059 12002067

[25] Che B, Shen S, Zhu Z, Wang A, Xu T, Peng Y, et al. Education Level and Long-term Mortality, Recurrent Stroke, and Cardiovascular Events in Patients With Ischemic Stroke. J Am Heart Assoc. 2020;9(16):e016671. doi: 10.1161/JAHA.120.016671 32779506 PMC7660803

[26] Jackson CA, Sudlow CLM, Mishra GD. Education, sex and risk of stroke: a prospective cohort study in New South Wales, Australia. BMJ Open. 2018;8(9):e024070. doi: 10.1136/bmjopen-2018-024070 30244216 PMC6157561

[27] Ariss RW, Minhas AMK, Lang J, Ramanathan PK, Khan SU, Kassi M, et al. Demographic and Regional Trends in Stroke-Related Mortality in Young Adults in the United States, 1999 to 2019. J Am Heart Assoc. 2022;11(18):e025903. doi: 10.1161/JAHA.122.025903 36073626 PMC9683653

[28] Trimble B, Morgenstern LB. Stroke in minorities. Neurol Clin. 2008;26(4):1177–90, xi. doi: 10.1016/j.ncl.2008.05.010 19026907 PMC2621018

[29] Cruz-Flores S, Rabinstein A, Biller J, Elkind MSV, Griffith P, Gorelick PB, et al. Racial-ethnic disparities in stroke care: the American experience: a statement for healthcare professionals from the American Heart Association/American Stroke Association. Stroke. 2011;42(7):2091–116. doi: 10.1161/STR.0b013e3182213e24 21617147

[30] Jenkins C, Ovbiagele B, Arulogun O, Singh A, Calys-Tagoe B, Akinyemi R, et al. Knowledge, attitudes and practices related to stroke in Ghana and Nigeria: A SIREN call to action. PLoS One. 2018;13(11):e0206548. doi: 10.1371/journal.pone.0206548 30444884 PMC6239297

[31] Admas M, Teshome M, Petrucka P, Telayneh AT, Alamirew NM. In-hospital mortality and its predictors among adult stroke patients admitted in Debre Markos Comprehensive Specialized Hospital, Northwest Ethiopia. SAGE Open Med. 2022. 10.1177/20503121221122465 PMC9459489 36093420

[32] Chang MC, Choo YJ, Seo KC, Yang S. The Relationship Between Dysphagia and Pneumonia in Acute Stroke Patients: A Systematic Review and Meta-Analysis. Front Neurol. 2022;13:834240. doi: 10.3389/fneur.2022.834240 35370927 PMC8970315

[33] Sadiq AO, Awotidebe AW, Saeys W, Truijen S, Wong TWL, Ng SSM, et al. Prevalence, associated factors and predictors of post stroke pneumonia in a Nigerian population: A retrospective study. J Stroke Cerebrovasc Dis. 2023;32(12):107404. doi: 10.1016/j.jstrokecerebrovasdis.2023.107404 37813084

[34] Akpalu J, Yawson AE, Osei-Poku F, Atiase Y, Yorke E, Adjei P, et al. Stroke Outcome and Determinants among Patients with and without Diabetes in a Tertiary Hospital in Ghana. Stroke Research and Treatment. 2018;2018:1–9. doi: 10.1155/2018/7521351 PMC6157204 30298101

[35] Price HL, Campbell SG. Isolated Lower Limb Weakness Following Hemorrhagic Stroke: A Case Report. Cureus. 2023;15(5):e38798. doi: 10.7759/cureus.38798 37303341 PMC10250139

