# Supplementary material for: In-hospital survival and predictors of mortality among stroke patients at a tertiary hospital in Ghana: A retrospective cohort study
Source: PLoS One. 2026 Jan 7;21(1):e0340447. doi: 10.1371/journal.pone.0340447 (PMC12795378; doi:10.1371/journal.pone.0340447)
Supplement: Attachment — Submitted filename: PONE-D-25-39162.pdf [file pone.0340447.s002.pdf]

PLOS ONE

PREDICTORS OF SURVIVAL OF STROKE PATIENTS IN THE TAMALE TEACHING HOSPITAL IN GHANA: A 3-YEAR RETROSPECTIVE REVIEW

--Manuscript Draft--

**Manuscript Number:**	PONE-D-25-39162
**Article Type:**	Research Article
**Full Title:**	PREDICTORS OF SURVIVAL OF STROKE PATIENTS IN THE TAMALE TEACHING HOSPITAL IN GHANA: A 3-YEAR RETROSPECTIVE REVIEW
**Short Title:**	PREDICTORS OF SURVIVAL OF STROKE PATIENTS IN THE TAMALE TEACHING HOSPITAL IN GHANA
**Corresponding Author:**	Sulemana Baba Abdulai<br>KNUST: Kwame Nkrumah University of Science and Technology<br>Tamale, Northern Region GHANA
**Keywords:**	Stroke, Survival analysis, Predictors, Mortality, Hemorrhagic stroke, Ischemic stroke.
**Abstract:**	Introduction: Stroke is one of the common causes of death and disability from non-communicable diseases worldwide. Ghana’s national estimates indicate a stroke prevalence of 7.96% and an incidence rate of 1.17%. The high incidence of stroke and related risk factors puts a significant burden on Ghana's healthcare system and is a major public health concern. This study aims to assess the predictors of survival among stroke patients admitted to the Tamale Teaching Hospital.<br>Methods: A retrospective electronic medical records review was conducted using data from the Lightwave Health Information Management System (LHIMS) and patients’ registry from January 1 2021 to December 31 2023, in the Tamale Teaching Hospital. Kaplan-Meier survival curve was used to determine the survival rate of stroke patients. Cox proportional hazard regression was conducted to determine the association between risk factors and survival status. Crude and adjusted hazard ratios along with their corresponding 95% confidence intervals, were presented. A p-value of 0.05 was considered statistically significant.<br>Results: A total of 998 stroke patients were included in the study, of which 39.4% died. The overall survival rate was 21% at the end of follow-up. The survival probability for female stroke patients was lower compared to their male counterparts. Female sex (AHR = 1.33, 95% CI: 1.07–1.65), belonging to the Dagomba ethnic group (AHR = 1.31, 95% CI: 1.05–1.62), co-morbidities like pneumonia (AHR = 1.59, 95% CI: 1.27–1.97), and diabetes mellitus (AHR = 0.62, 95% CI: 0.47–0.82), systolic blood pressure ≥ 130 mmHg (AHR = 1.35, 95% CI: 1.06 – 1.73), and temperature ≥ 37.5 °C (AHR = 1.79, 95% CI: 1.37 – 2.33) were predictors of mortality in stroke patients.<br>Conclusion: The study concluded that female stroke patients experienced higher mortality rates and lower survival rates compared to males. The identified mortality predictors underscore the importance of focused interventions to enhance survival outcomes for stroke patients at TTH.
**Order of Authors:**	Sulemana Baba Abdulai
	Julius Kwabena Karikari
	Penias Tembo
	Theogene Habumugisha
	William Tembo
	Alhaji Ibrahim Cobbinah
	Yeetey Enuameh
**Additional Information:**	
**Question**	**Response**
**Financial Disclosure**	The author(s) received no specific funding for this work.

Powered by Editorial Manager® and ProduXion Manager® from Aries Systems Corporation

Enter a financial disclosure statement that describes the sources of funding for the work included in this submission. Review the submission guidelines for detailed requirements. View published research articles from *PLOS ONE* for specific examples.<br><br>This statement is required for submission and **will appear in the published article** if the submission is accepted. Please make sure it is accurate.<br><br>**Funded studies**<br>Enter a statement with the following details:<br>• Initials of the authors who received each award<br>• Grant numbers awarded to each author<br>• The full name of each funder<br>• URL of each funder website<br>• Did the sponsors or funders play any role in the study design, data collection and analysis, decision to publish, or preparation of the manuscript?<br><br>Did you receive funding for this work?	
**Competing Interests**<br><br>Use the instructions below to enter a competing interest statement for this submission. On behalf of all authors, disclose any competing interests that could be perceived to bias this work—acknowledging all financial support and any other relevant financial or non-financial competing interests.<br><br>This statement is required for submission and **will appear in the published article** if the submission is accepted. Please make sure it is accurate and that any funding sources listed in your Funding Information later in the submission form are also declared in your Financial Disclosure statement.<br><br>View published research articles from	The authors have declared that no competing interests exist.

Powered by Editorial Manager® and ProduXion Manager® from Aries Systems Corporation

PLOS ONE for specific examples.<br><br>**NO authors have competing interests**<br><br>Enter: *The authors have declared that no competing interests exist.*<br><br>**Authors with competing interests**<br><br>Enter competing interest details beginning with this statement:<br><br>*I have read the journal's policy and the authors of this manuscript have the following competing interests: [insert competing interests here]*<br><br>* typeset	
**Ethics Statement**<br><br>Enter an ethics statement for this submission. This statement is required if the study involved:<br><br>• Human participants<br>• Human specimens or tissue<br>• Vertebrate animals or cephalopods<br>• Vertebrate embryos or tissues<br>• Field research<br><br>Write "N/A" if the submission does not require an ethics statement.<br><br>General guidance is provided below. Consult the submission guidelines for detailed instructions. **Make sure that all information entered here is included in the Methods section of the manuscript.**	Ethical approval for the study was obtained from the Committee on Human Research, Publications and Ethics at the Kwame Nkrumah University of Science and Technology (KNUST), under approval number CHRPE/AP/237/24. Given the retrospective nature of the study, the requirement for informed consent was waived.

**Format for specific study types**<br><br>**Human Subject Research (involving human participants and/or tissue)**<br>• Give the name of the institutional review board or ethics committee that approved the study<br>• Include the approval number and/or a statement indicating approval of this research<br>• Indicate the form of consent obtained (written/oral) or the reason that consent was not obtained (e.g. the data were analyzed anonymously)<br><br>**Animal Research (involving vertebrate animals, embryos or tissues)**<br>• Provide the name of the Institutional Animal Care and Use Committee (IACUC) or other relevant ethics board that reviewed the study protocol, and indicate whether they approved this research or granted a formal waiver of ethical approval<br>• Include an approval number if one was obtained<br>• If the study involved *non-human primates*, add *additional details* about animal welfare and steps taken to ameliorate suffering<br>• If anesthesia, euthanasia, or any kind of animal sacrifice is part of the study, include briefly which substances and/or methods were applied<br><br>**Field Research**<br><br>Include the following details if this study involves the collection of plant, animal, or other materials from a natural setting:<br>• Field permit number<br>• Name of the institution or relevant body that granted permission	
**Data Availability**<br><br>Authors are required to make all data underlying the findings described fully available, without restriction, and from the time of publication. PLOS allows rare exceptions to address legal and ethical concerns. See the PLOS Data Policy and FAQ for detailed information.	Yes - all data are fully available without restriction

A Data Availability Statement describing where the data can be found is required at submission. Your answers to this question constitute the Data Availability Statement and **will be published in the article**, if accepted.<br><br>**Important:** Stating 'data available on request from the author' is not sufficient. If your data are only available upon request, select 'No' for the first question and explain your exceptional situation in the text box.<br><br>Do the authors confirm that all data underlying the findings described in their manuscript are fully available without restriction?	
**Describe where the data may be found in full sentences. If you are copying our sample text, replace any instances of XXX with the appropriate details.**<br><br>• If the data are **held or will be held in a public repository**, include URLs, accession numbers or DOIs. If this information will only be available after acceptance, indicate this by ticking the box below. For example: *All XXX files are available from the XXX database (accession number(s) XXX, XXX.).*<br>• If the data are all contained **within the manuscript and/or Supporting Information files**, enter the following: *All relevant data are within the manuscript and its Supporting Information files.*<br>• If neither of these applies but you are able to provide **details of access elsewhere**, with or without limitations, please do so. For example:<br><br>*Data cannot be shared publicly because of [XXX]. Data are available from the XXX Institutional Data Access / Ethics Committee (contact via XXX) for researchers who meet the criteria for access to confidential data.*<br><br>*The data underlying the results presented in the study are available from (include the name of the third party*	The anonymised datasets used and/or analysed during the current study are not publicly accessible; however, they can be obtained from the corresponding author upon reasonable request.

and contact information or URL).<br>• This text is appropriate if the data are owned by a third party and authors do not have permission to share the data.<br><br>* typeset	
Additional data availability information:	

Manuscript

Click here to access/download;Manuscript;MANUSCRIPT_PLOS_ONE.doc

PREDICTORS OF SURVIVAL OF STROKE PATIENTS IN THE TAMALE TEACHING HOSPITAL IN GHANA: A 3-YEAR RETROSPECTIVE REVIEW

Sulemana Baba Abdulai[1*], Julius Kwabena Karikari[1,2], Penias Tembo[3,4], Theogene Habumugisha[5], William Tembo[6], Alhaji Ibrahim Cobbinah[1], Yeetey Enuameh[1]

Affiliations

[1] Department of Epidemiology and Biostatistics, School of Public Health, Kwame Nkrumah University of Science and Technology, Kumasi, Ghana.

[2] University Hospital, Kwame Nkrumah University of Science and Technology, Kumasi, Ghana.

[3] Department of Epidemiology and Biostatistics, Arnold School of Public Health, University of South Carolina, Columbia SC, USA.

[4] Cancer Prevention and Control Program, University of South Carolina, Columbia SC, USA.

[5] Department of Global Public Health and Primary Care, Center for International Health, University of Bergen, Bergen, Norway.

[6] Department of Internal Medicine, Neurology Division, University Teaching Hospital, Lusaka, Zambia

***Corresponding Author:**

Sulemana Baba Abdulai

babasulemanaabdulai@gmail.com

Abstract

Introduction: Stroke is one of the common causes of death and disability from non-communicable diseases worldwide. Ghana's national estimates indicate a stroke prevalence of 7.96% and an incidence rate of 1.17%. The high incidence of stroke and related risk factors puts a significant burden on Ghana's healthcare system and is a major public health concern. This study aims to assess the predictors of survival among stroke patients admitted to the Tamale Teaching Hospital.

Methods: A retrospective electronic medical records review was conducted using data from the Lightwave Health Information Management System (LHIMS) and patients' registry from January 1 2021 to December 31 2023, in the Tamale Teaching Hospital. Kaplan-Meier survival curve was used to determine the survival rate of stroke patients. Cox proportional hazard regression was conducted to determine the association between risk factors and survival status. Crude and adjusted hazard ratios along with their corresponding 95% confidence intervals, were presented. A p-value of 0.05 was considered statistically significant.

Results: A total of 998 stroke patients were included in the study, of which 39.4% died. The overall survival rate was 21% at the end of follow-up. The survival probability for female stroke patients was lower compared to their male counterparts. Female sex (AHR = 1.33, 95% CI: 1.07–1.65), belonging to the Dagomba ethnic group (AHR = 1.31, 95% CI: 1.05–1.62), co-morbidities like pneumonia (AHR = 1.59, 95% CI: 1.27–1.97), and diabetes mellitus (AHR = 0.62, 95% CI: 0.47–0.82), systolic blood pressure ≥ 130 mmHg (AHR = 1.35, 95% CI: 1.06 – 1.73), and temperature ≥ 37.5 °C (AHR = 1.79, 95% CI: 1.37 – 2.33) were predictors of mortality in stroke patients.

Conclusion: The study concluded that female stroke patients experienced higher mortality rates and lower survival rates compared to males. The identified mortality predictors underscore the importance of focused interventions to enhance survival outcomes for stroke patients at TTH.

Keywords: Stroke, Survival analysis, Predictors, Mortality, Hemorrhagic stroke, Ischemic stroke.

Introduction

Stroke, as defined by the World Health Organization (WHO), is a sudden disruption in brain function lasting more than a day or leading to death, primarily caused by vascular issues [1]. It is a multifaceted disease influenced by risk factors such as hypertension, diabetes, and heart failure [2]. Globally, stroke remains a leading cause of death and disability, responsible for significant health burdens, including an annual loss of approximately 1,484 disability-adjusted life years (DALYs) per 100,000 people [3]. Survivors often endure long-term physical, cognitive, and emotional challenges, imposing substantial demands on families, social, and healthcare systems [4].

In sub-Saharan Africa (SSA), the burden of stroke is rapidly increasing due to an epidemiological shift towards non-communicable diseases (NCDs) [5]. Stroke incidence and mortality rates in SSA are alarmingly high, with incidence reaching up to 1,460 cases per 100,000 people, and a three-year fatality rate exceeding 80% [6]. Challenges such as inadequate health infrastructure, limited neurologists, and low awareness of stroke symptoms exacerbate poor outcomes in the region [7]. In Ghana, stroke ranks among the top five causes of death, with concerning trends in rising admissions and a one-month hospital fatality rate of up to 43% [8,9]. Over the past three decades, the country has witnessed a consistent rise in stroke incidence, admissions, and mortality. For instance, stroke-related admissions in Kumasi increased from 5.32 per 1,000 in 1983 to 13.85 per 1,000 in 2010, with one-month fatality rates reaching 41% in the Central Region [9]. Key modifiable risk factors, including high salt intake, physical inactivity, and limited consumption of green leafy vegetables, are prevalent in Ghana [10]. Additionally, younger populations under 40 years are increasingly affected, posing significant socio-economic challenges [11].

The Tamale Teaching Hospital (TTH) in Northern Ghana, a major referral centre, reports high stroke-related mortality, with stroke ranking as the third leading cause of death in its Accident and Emergency Department [9]. Factors such as delayed presentation, socio-cultural barriers to healthcare, and resource constraints contribute to poor stroke outcomes [9]. While stroke-related mortality has been extensively studied, limited data exist on survival and its determinants, particularly in the Ghana context [[illegible]

Despite significant advancements in stroke care, limited region-specific data on stroke survival and its determinants impede the development of targeted interventions in Ghana. Current research has predominantly focused on stroke mortality, with few studies on survival outcomes and their predictors [12]. This knowledge gap hinders the development of evidence-based stroke management strategies in LMICs, including the establishment of dedicated stroke units, the availability of diagnostic tools such as MRI machines, and the training of healthcare professionals essential for improving stroke care [13–15]. This study aims to address these gaps by analysing predictors of survival of stroke patients in the Tamale Teaching Hospital: a 3-Year retrospective study. By examining patient demographics, clinical characteristics, and treatment outcomes, this study aims to provide a region-specific understanding of stroke patterns and interventions. The findings aim to inform healthcare planning, enhance resource allocation, and support the development of data-driven public health policies in the Northern Region. Furthermore, the study aligns with WHO's Agenda 2030 for Sustainable Development, particularly its goal of reducing mortality from major NCDs by one-third by 2030.

Materials and methods

Patient identification

We conducted a retrospective review of electronic medical records using the Lightwave Health Information Management System (LHIMS) to identify all patients admitted with a diagnosis of stroke at the Tamale Teaching Hospital (TTH) between January 1, 2021, and December 31, 2023. Medical records were accessed for research purposes between April 15 and June 15, 2024. The study included patients aged 18 years and above who were diagnosed with either ischemic or hemorrhagic stroke during the study period. Patients were excluded if their medical records were incomplete or missing, or if the type of stroke was not specified as either ischemic or hemorrhagic (Figure 1). The authors had no access to information that could identify individual participants at any point during or after data collection. All data used for the study were fully anonymised prior to analysis to ensure the privacy and confidentiality of patients. Ethical approval for the study was obtained from the Committee on Human Research, Publications and Ethics at the Kwame Nkrumah University of Science and Technology (KNUST), under approval number CHRPE/AP/237/24. Given the retrospective nature of the study, the requirement for informed consent was waived.

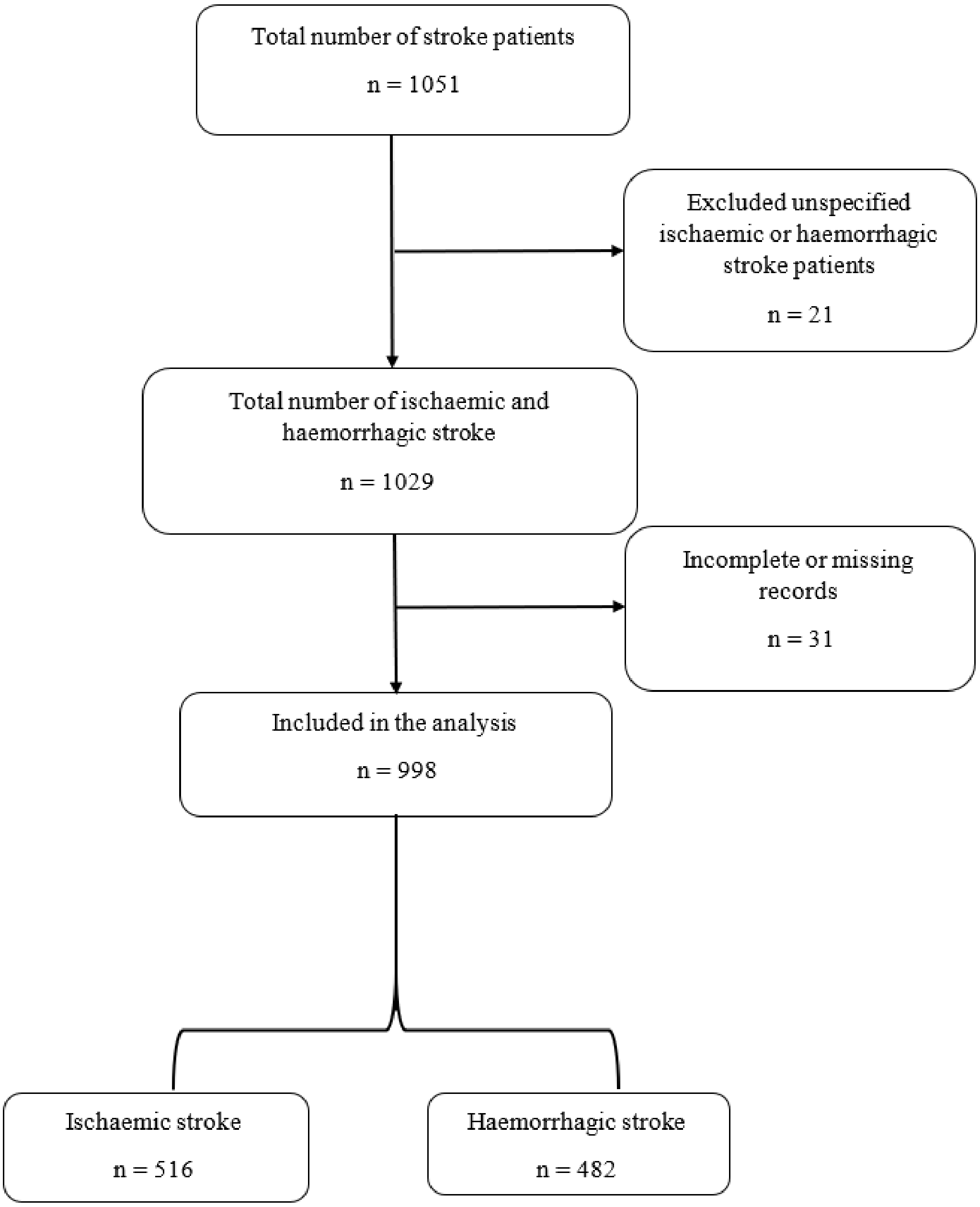

Figure 1: Identification and eligibility criteria used for the study population in the analysis

Study Variables

The outcome variable, survival status, was categorised based on the discharge diagnosis as either dead or alive. The identified predictors were categorised into two sections which included; demographic/baseline characteristics (age, gender,

educational level, occupation, ethnicity, family history of stroke, smoking status, alcohol intake status, body temperature, systolic blood pressure (BP), diastolic BP, heart rate, respiratory rate, and Glasgow Coma Score (GCS) level) and clinical characteristics (family medical history, presence of hypertension, diabetes, kidney disease, occurrence of pneumonia, stroke type, atrial fibrillation, right upper limb weakness, left upper limb weakness, seizures, aphasia, dysarthria, and dysphagia) (Table 1). Also, some important variables like the date of admission and date of discharge/death were collected to compute the length of hospital stay for a patient.

Data Analysis

Data extracted from LHIMS was entered into Excel, cleaned, and subsequently exported to STATA V.17 for analysis. Univariable descriptive statistics, including frequencies and percentages, were presented in tables, with means and standard deviations for normally distributed continuous variables, and medians with interquartile ranges for skewed variables. All continuous variables were assessed for normality using the Shapiro–Wilk test, and their distributions were visualised using histograms. Kaplan-Meier survival curves were used to estimate average survival time and overall survival rates. A log-rank test was performed to assess the difference between sex (male and female).

A stepwise Cox proportional hazards regression model was applied to identify variables associated with survival outcomes. Hazard ratios were used to measure the strength of these associations. The Schoenfeld residual test was conducted to verify the proportional hazards assumption, with variables meeting a p-value > 0.05 considered to satisfy the assumption. A bivariable Cox proportional hazards model was fitted for all predictors and variables with a p-value of less than 0.25 in order not to leave out important variables that may influence the outcome, and these were selected for inclusion in the stepwise multivariable Cox regression model. Multicollinearity among the predictors included in the multivariable model was assessed using the Variance Inflation Factor (VIF), and predictors with a VIF < 5 were included in the final model. [Mean VIF = 1.64, Max VIF = 2.63, Min VIF = 1.05], indicating no significant multicollinearity among the predictors. Statistical significance was set at p-value < 0.05 and a 95% confidence interval (CI).

Results

Demographic/baseline and clinical characteristics of stroke patients at TTH

Most patients were aged between 45 and 65 years (44.19%). More than half of the patients were male (54.71%). Regarding occupation, most were employed (61.72%). Majority had no formal education (70.64%). Ethnically, Dagombas represented the largest group (52.20%). Most patients reported no family history of stroke (96.79%), were non-smokers (93.89%), and did not consume alcohol (88.38%). Clinically, most patients presented with a temperature below 37.5 °C (86.07%) and had systolic and diastolic blood pressures of ≥130 mmHg (68.84%) and ≥80 mmHg (68.74%), respectively. A heart rate above 100 bpm was observed in 62.83% of patients, while 54.91% had a respiratory rate of 20 or fewer breaths per minute. The majority of patients had a Glasgow Coma Scale (GCS) score of ≥9 (72.55%). Hypertension was the most common, affecting 78.56% of patients, followed by diabetes (21.14%). Only a small proportion had kidney disease (2.30%). Pneumonia was documented in 36.37% of cases. Ischemic stroke was slightly more common than haemorrhagic stroke, accounting for 51.70% and 48.30% of cases, respectively. Atrial fibrillation was rare, reported in only 0.30% of patients. Regarding motor function and neurological deficits, the most common presentation was right upper limb weakness (32.67%), followed by left upper limb weakness (28.86%). Right lower limb weakness was observed in 35.77% of patients, while left lower limb weakness occurred in 32.46%. Seizures were noted in 12.93% of patients. Aphasia was present in 34.57% of cases, dysarthria in 15.03%, and dysphagia in 14.93% (Table 1). Approximately 60.62% of patients survived their admission, while 39.38% died during hospitalisation (Figure 2).

Table 1: Demographic/baseline and clinical characteristics of stroke patients at TTH

Characteristics	Frequency (N = 998)	Percentage (%)
**Age**		
< 45	178	17.84
45-65	441	44.19
≥ 66	379	37.98
**Sex**		
Male	546	54.71
Female	452	45.29
**Occupation**		
Unemployed	281	28.16
Employed	616	61.72
Retired	101	10.12
**Educational status**		
No education	705	70.64
Basic	40	4.01
SHS	105	10.52
Tertiary	148	14.83
**Ethnicity**		
Dagombas	521	52.20
Gonjas	35	3.51
Others[1]	442	44.29
**Family history of stroke**		
No	966	96.79
Yes	32	3.21
**Smoking**		
No	937	93.89
Yes	61	6.11
**Alcohol intake**		
No	882	88.38
Yes	116	11.62
**Temperature (°C)**		
< 37.5	859	86.07
≥ 37.5	139	13.93
**Systolic BP (mmHg)**		
< 130	311	31.16
≥ 130	687	68.84
**Diastolic BP (mmHg)**		
< 80	312	31.26
≥ 80	686	68.74
**Heart rate (bpm)**		
≤ 100	371	37.17
> 100	627	62.83
**Respiratory rate (breaths/min)**		
≤ 20	548	54.91
> 20	450	45.09
**GCS level**		
< 9	274	72.55
≥ 9	724	27.45

Characteristics	Frequency (N = 998)	Percentage (%)
**Hypertension**		
No	214	21.44
Yes	784	78.56
**Diabetes**		
No	787	78.86
Yes	211	21.14
**Kidney disease**		
No	975	97.70
Yes	23	2.30
**Pneumonia**		
No	635	63.63
Yes	363	36.37
**Stroke type**		
Ischemic	516	51.70
Haemorrhagic	482	48.30
**Atrial fibrillation**		
No	995	99.70
Yes	3	0.30
**Right upper limb weakness**		
No	672	67.33
Yes	326	32.67
**Left upper limb weakness**		
No	710	71.14
Yes	288	28.86
**Right lower limb weakness**		
No	641	64.23
Yes	357	35.77
**Left lower limb weakness**		
No	674	67.54
Yes	324	32.46
**Seizures**		
No	869	87.07
Yes	129	12.93
**Aphasia**		
No	653	65.43
Yes	345	34.57
**Dysarthria**		
No	848	84.97
Yes	150	15.03
**Dysphagia**		
No	849	85.07
Yes	149	14.93
**Urea (mmol/L)**		
Median (IQR)	5.9 (4.82)	
**Creatinine(µmolL)**		
Median (IQR)	82.6 (60.30)	
**Bilirubin (µmolL)**		
Median (IQR)	11.5 (11.99)	
**Random blood sugar (mmol/L)**		

Characteristics	Frequency (N = 998)	Percentage (%)
Median (IQR)	7.7(3.20)	

1 = Wala, Kusasi, Frafra

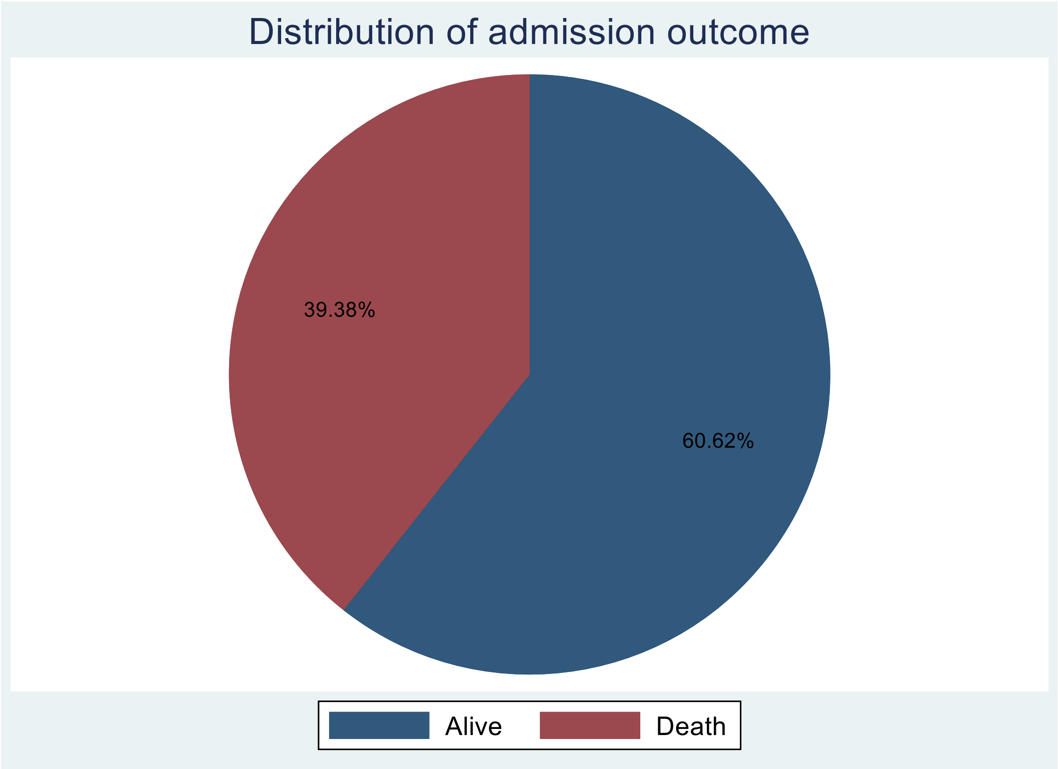

Figure 2: Admission outcome of stroke patients at TTH

Survival Probability of Stroke Patients

Figure 3 presents the Kaplan-Meier survival curve for stroke patients. The overall survival probability was 21.0%, with a mean survival time of 67 days (95% CI: 42 – 91). The curve also shows a decline in survival rates with longer hospital stays. The probability of survival was 90.0% on the 1st day, 71.0% on the 5th day, and 60.0% on the 10th day. By the end of the 50-day observation period, although some patients continued to survive, the survival probability had dropped significantly to 42.5%.

Throughout the follow-up period, males consistently demonstrate a higher survival probability than females. The survival rate for females declined sharply, indicating a higher mortality rate over the same timeframe (Figure 4). For instance, on the 5th day,

the survival probability for females was 68.0%, compared to 73.0% for males, and on the 10th day, it was 64.0% for males and 55.0% whereas for females. The median survival time for females was 13 days (95% CI: 10 – 31), while for males, it was 17 days.

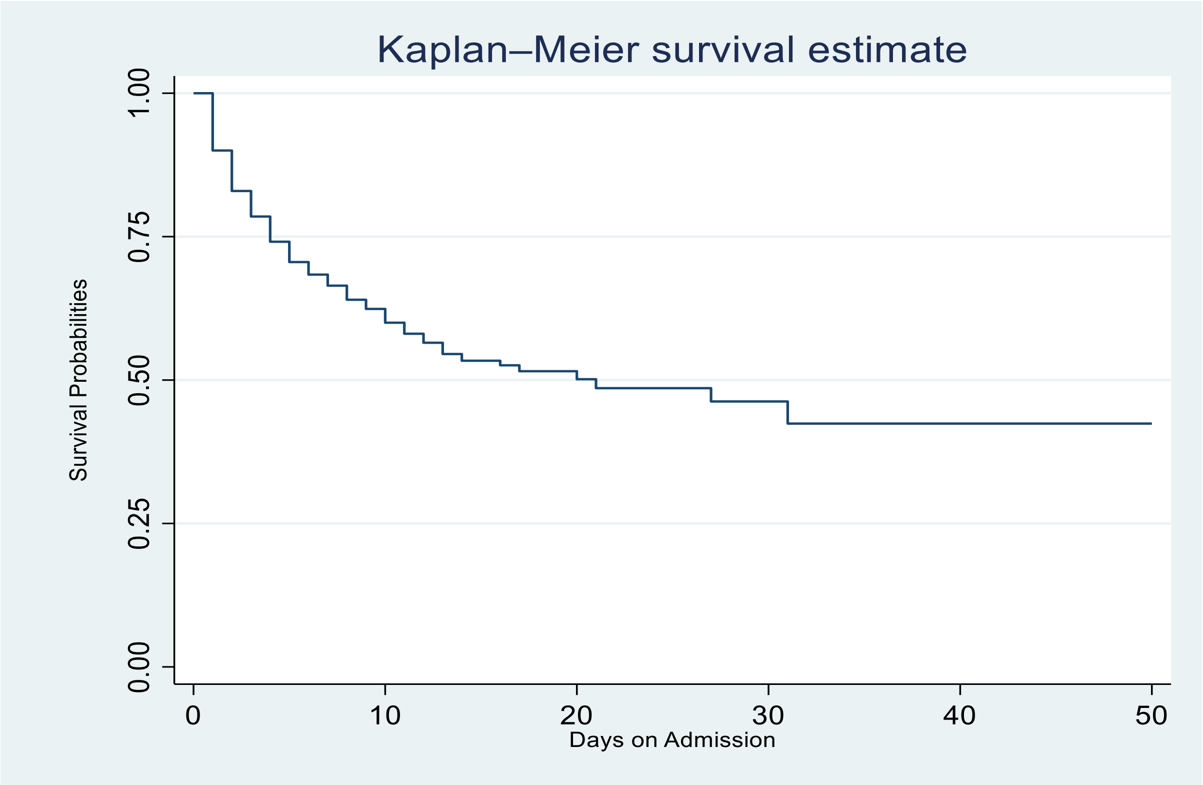

Figure 3: Overall Kaplan-Meier survival curve of stroke patients at TTH.

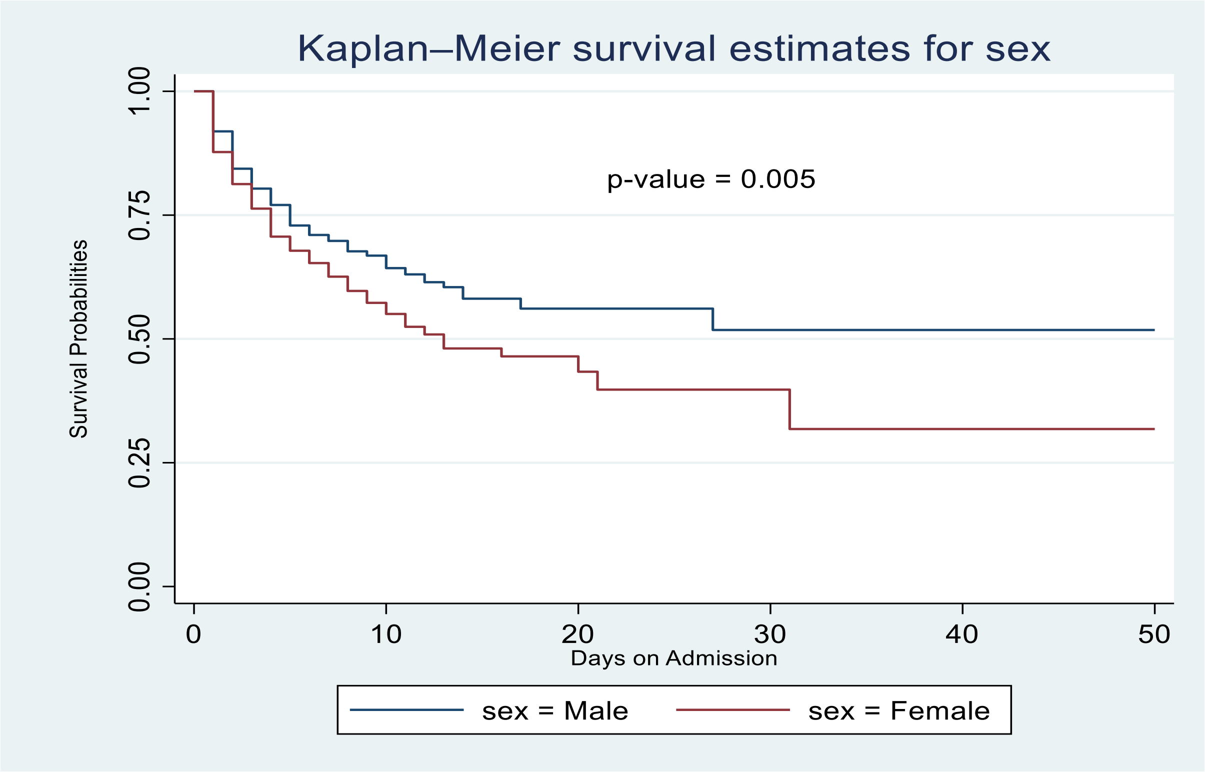

Figure 4: Kaplan-Meier survival analysis by sex

Sociodemographic and Clinical Factors Associated with the Survival Rate of Individuals Diagnosed with Stroke

Patients aged 45–65 years had a significantly higher hazard of death compared to those younger than 45 years (AHR = 1.28; 95% CI: 1.03–1.58). Female patients had a significantly 33% higher risk of mortality compared to males (AHR = 1.33; 95% CI: 1.07–1.65). Educational status was also significantly associated with survival. Patients with basic education had a 54% lower risk of death compared to those with no education. Among ethnic groups, Dagombas had a 31% increased risk of death compared to other groups (AHR = 1.31; 95% CI: 1.05–1.62). Patients with diabetes had a 39% lower risk of mortality compared to non-diabetics (AHR = 0.62; 95% CI: 0.47–0.82). Similarly, individuals presenting with right lower limb weakness had a 33% lower risk of death than those without the condition (AHR = 0.67; 95% CI: 0.53–0.85). Patients with elevated temperature (≥ 37.5 °C) had a 79% higher hazard of death

(AHR = 1.79; 95% CI: 1.37–2.33), while those with pneumonia had a 1.59 times higher risk of mortality than patients without pneumonia (AHR = 1.59; 95% CI: 1.27–1.97). Additionally, patients with systolic blood pressure ≥ 130 mmHg exhibited a 32% higher risk of death compared to those with lower systolic pressure (AHR = 1.35, 95% CI: 1.06 – 1.73). Interestingly, a heart rate above 100 bpm was associated with a significantly lower risk of mortality (AHR = 0.50, 95% CI: 0.40–0.62), as was a respiratory rate above 20 (AHR = 0.63, 95% CI: 0.49–0.79). Higher oxygen saturation was significantly associated with better survival (AHR = 0.96, 95% CI: 0.95–0.98), while for each mmol/L increase in urea, the risk of mortality increased by 2% (AHR = 1.02; 95% CI: 1.01–1.03 per mmol/L) (Table 2).

Table 2: Analysis of sociodemographic and clinical factors associated with the survival rate of patients diagnosed with stroke

Characteristics	Crude HR	95% CI	p-value	Adjusted HR	95% CI	p-value
**Age group**						
< 45	Ref			Ref		
45-65	1.25	0.92-1.70	0.154	1.28	1.03-1.58	0.027
≥ 66	1.07	0.78-1.48	0.663			
**Sex**						
Male	Ref			Ref		
Female	1.34	1.08-1.66	0.008	1.33	1.07-1.65	0.010
**Educational status**						
No education	Ref			Ref		
Basic	0.49	0.24-0.98	0.044	0.46	0.23-0.93	0.039
SHS	0.95	0.67-1.36	0.669			
Tertiary	0.57	0.40-0.83	0.003			
**Ethnicity**						
Others[1]	Ref			Ref		
Gonjas	0.62	0.29-1.33	0.219			
Dagombas	1.34	1.08-1.67	0.008	1.31	1.05-1.62	0.018
**Alcohol intake**						
No	Ref			Ref		
Yes	0.75	0.52-1.07	0.113	0.69	0.48-0.99	0.044
**Temperature (°C)**						
< 37.5	Ref			Ref		
≥ 37.5	2.26	1.75-2.91	<0.001	1.79	1.37-2.33	<0.001
**Systolic BP (mmHg)**						
< 130	Ref			Ref		
≥ 130	1.45	1.14-1.85	0.003	1.35	1.06-1.73	0.017
**Diastolic BP (mmHg)**						
< 80	Ref					
≥ 80	1.45	1.13 -1.85	0.003			

Characteristics	Crude HR	95% CI	p-value	Adjusted HR	95% CI	p-value
**Heart rate (bpm)**						
≤ 100	Ref			Ref		
> 100	0.40	0.32-0.49	<0.001	0.52	0.42-0.66	<0.001
**Respiratory rate (breaths/min)**						
< 20	Ref			Ref		
≥ 20	0.51	0.41-0.64	<0.001	0.60	0.47-0.76	<0.001
**GCS level**						
≥ 9	Ref					
< 9	1.23	0.97-1.53	0.094			
**Diabetes**						
No	Ref			Ref		
Yes	0.73	0.55-0.95	0.021	0.62	0.47-0.82	0.001
**Pneumonia**						
No	Ref			Ref		
Yes	1.89	1.53-2.34	<0.001	1.59	1.27-1.97	<0.001
**Stroke type**						
Ischaemic	Ref					
Haemorrhagic	1.17	0.95-1.45	0.144			
**Right upper limb weakness**						
No	Ref					
Yes	0.76	0.60-0.96	0.025			
**Right lower limb weakness**						
No	Ref					
Yes	0.72	0.57-0.91	0.005	0.67	0.53-0.85	0.001
**Dysarthria**						
No	Ref					
Yes	0.69	0.49-0.96	0.030			
**Urea (mmol/L)**						
	1.02	1.01-1.03	<0.001	1.02	1.01-1.03	<0.001
**Creatinine (µmolL)**						
	1.00	1.00-1.00	<0.001			

Characteristics	Crude HR	95% CI	p-value	Adjusted HR	95% CI	p-value
**Bilirubin (µmolL)**	1.01	0.99-1.02	0.196			
**Random blood sugar (mmol/L)**	1.01	1.00-1.02	0.022			

HR = Hazard ratio, Ref = reference group, CI =Confidence interval, 1 = Wala, Kusasi, Frafra

Discussion

We identified several significant demographic and clinical predictors of stroke survival. Female sex, Dagomba ethnicity, elevated temperature, pneumonia, higher systolic blood pressure, and increased urea levels were associated with higher mortality risk. Conversely, basic education, diabetes, right lower limb weakness, elevated heart and respiratory rates, and higher oxygen saturation were linked to improved survival outcomes.

The overall survival probability was high in the initial days of admission but decreased as follow-up time increased. Revealing further that a high number of patients died in the first 10 days after being diagnosed with stroke. This is comparable to a study in North West Ethiopia, with similar observations [4]. Also, the median survival time of 21 days was lower than in a study in Ethiopia that had a median survival time of 41 days [16].

Males consistently showed a higher survival probability throughout the follow-up period, indicating a gender disparity in stroke outcomes. This aligns with findings from a study by [17] conducted in South Korea. The steep decline in survival rates among females, indicates a higher mortality rate, evident as early as the 5th-day post-stroke, where the survival probability for females was 68.0% compared to 73.0% for males. This pattern persists, with a significant gap by the 10th day, as survival probabilities drop to 55.0% for females and 64.0% for males. The median survival time further underscores this difference, with females showing a notably shorter median survival time of 13 days compared to 17 days for males. This disparity may be attributed to more severe post-stroke complications or other factors impacting female survival rates over time [18].

The findings also revealed that the odds of mortality were high among females compared to males. This aligns with a study in Ghana at the Korlebu Teaching Hospital [19]. Several factors may contribute to the higher burden of stroke-related mortality and disability among females. Sociocultural gender roles and biological differences influence stroke risk, assessment, treatment, and outcomes. The relationship between general stroke risk factors and female-specific risk variables differs considerably. Additionally, there are differences in how women experience stroke symptoms, respond to treatment, and recover after a stroke compared to men [18].

The study also revealed that stroke patients with no formal education faced a higher risk of mortality compared to those with education. Specifically, having a basic education was associated with approximately a 54% lower risk of death. This finding is consistent with studies conducted in Ghana and China [12,20]. This finding may be attributed to individuals with no education often displaying less healthy lifestyle behaviours and greater clinical risk factors for stroke, a pattern observed in both genders [21].

Also, the study found that individuals of Dagomba ethnicity had a higher risk of mortality following stroke. While direct comparisons are limited, this observation aligns with findings from the United States, where studies have consistently reported higher stroke mortality rates among Black populations [22,23]. A possible explanation is that cultural beliefs within certain ethnic groups may encourage reliance on traditional healing practices, leading to delays in seeking hospital care [24,25]. These disparities include a lack of awareness about stroke symptoms, delays in seeking timely treatment, and limited understanding of risk factors. Differences in attitudes, beliefs, and adherence to medical advice also vary across races and ethnicities.

The study revealed that pneumonia was also a predictor of mortality. The risk of mortality was high among stroke patients with pneumonia complications. This is consistent with a study in Ghana, Nigeria and Ethiopia [12,16,26]. The increased risk of mortality can be linked to several factors. Post-stroke patients frequently encounter swallowing difficulties and limited mobility, both of which can contribute to the development of lung infections like pneumonia [27]. Moreover, strokes can induce an immune response that heightens vulnerability to infections, disrupts the tracheal epithelium, diminishes lung clearance, and hampers the expulsion of secretions, further elevating the likelihood of pneumonia [28].

Interestingly, the study revealed that stroke patients with diabetes had a lower risk of mortality, which contrasts with findings observed in Ghana [12,29]. This finding needs to be further investigated as it is very counterintuitive unless something special is being done in the population or the health facility within which these cases are being picked

Furthermore, we found that patients presenting with right lower limb weakness had a lower risk of death compared to those without such weakness. Although this specific finding has limited direct support in existing literature, a study conducted in Canada reported that outcomes tend to be more favourable in individuals with motor strokes, particularly those presenting with monoparesis, defined as weakness in a single limb [30]. While this does not directly align with our findings, it suggests that isolated motor deficits may be associated with less severe stroke presentations, potentially contributing to improved survival. Overall, patients with motor stroke symptoms often experience fewer complications and shorter hospital stays, which may partly explain the observed association in our study. Also, these patients are more likely to experience symptom resolution by the time of hospital discharge, which further

supports the observation that isolated motor deficits may predict better prognoses compared to more extensive motor involvement.

Implications for practice and research

The identification of both demographic and clinical predictors of stroke mortality has important implications for clinical practice. High-risk patients, such as females, individuals with low or no education, those presenting with aphasia, pneumonia, elevated temperature, or high systolic blood pressure, may benefit from closer monitoring and aggressive management during the acute phase of stroke. The protective associations observed with diabetes suggest potential responsiveness to treatment that warrants further investigation. Early identification and prompt intervention for modifiable risk factors like diabetes could improve survival outcomes, especially during the critical first 10 days post-admission when mortality risk is highest.

Strengths and Limitations

This study provides valuable insights into the factors that influence survival among stroke patients in a real-world clinical setting. By using a multivariable Cox regression model, we were able to control for several demographic and clinical variables, helping us identify the key predictors of mortality more accurately. The study also benefits from a relatively large sample size and a three-year follow-up period, which strengthens the reliability of our findings. Importantly, by examining both social and clinical factors, we offer a more holistic picture of the challenges stroke patients face in our context.

That said, our study has a few limitations worth noting. Because it is based on retrospective data, we were limited to the information recorded in patients' medical files, which were sometimes incomplete or missing key details. We also could not assess stroke severity scores or functional outcomes at admission, which might have

further clarified the survival patterns we observed. In addition, we only looked at what happened during the hospital stay, so we might have missed deaths that occurred after discharge.

Conclusion

The study revealed that stroke patients treated at the Tamale Teaching Hospital face a declining probability of survival over time, with the highest risk of death occurring within the first 10 days of admission. Survival also appeared to decrease with prolonged hospital stay, potentially reflecting the severity of patients' conditions. Key factors associated with increased mortality included pneumonia, being female, elevated body temperature ($\geq 37.5\,°C$), high systolic blood pressure (≥ 130 mmHg), and belonging to the Dagomba ethnic group.

Acknowledgement

We acknowledge the Senior Health Information Officer at TTH, Mr. Abdul–Hafiz Zakari, for his generous assistance and cooperation during the research.

Data availability

The anonymised datasets used and/or analysed during the current study are not publicly accessible; however, they can be obtained from the corresponding author upon reasonable request.

Authors' contributions

SBA, JKK, PT, TH, WT, AIC and YE contributed as follows: SBA and YE conceptualised the study and developed the methodology; SBA collected the data; SBA and JKK performed formal analysis; SBA drafted the initial manuscript; JKK, PT, TH, WT, AIC, and YE critically reviewed and edited the manuscript. YE supervised the

study. All authors have read and approved the final manuscript and agree to be accountable for all aspects of the work.

Competing interests

The authors declare no competing interests.

Funding

The authors had no support or funding for this current study.

References

[1] Hatano S. Experience from a multicentre stroke register: a preliminary report. 1976;

[2] Russell JBW, Charles E, Conteh V, Lisk DR. Risk factors, clinical outcomes and predictors of stroke mortality in Sierra Leoneans: A retrospective hospital cohort study. Ann Med Surg. 2020 Nov 4;60:293–300.

[3] Feigin VL, Stark BA, Johnson CO, Roth GA, Bisignano C, Abady GG, et al. Global, regional, and national burden of stroke and its risk factors, 1990–2019: a systematic analysis for the Global Burden of Disease Study 2019. Lancet Neurol. 2021 Oct;20(10):795–820.

[4] Walelgn N, Abyu GY, Seyoum Y, Habtegiorgis SD, Birhanu MY. The Survival Status and Predictors of Mortality Among Stroke Patients at North West Ethiopia. Risk Manag Healthc Policy. 2021 Jul 14;14:2983–94.

[5] Ayehu GW, Yitbarek GY, Jemere T, Chanie ES, Feleke DG, Abebaw S, et al. Case fatality rate and its determinants among admitted stroke patients in public referral hospitals, Northwest, Ethiopia: A prospective cohort study. PLoS ONE. 2022 Sep 15;17(9):e0273947.

[6] Akinyemi RO, Ovbiagele B, Adeniji OA, Sarfo FS, Abd-Allah F, Adoukonou T, et al. Stroke in Africa: profile, progress, prospects and priorities. Nat Rev Neurol. 2021;17(10):634–56.

[7] Sarfo FS, Akpa OM, Ovbiagele B, Akpalu A, Wahab K, Obiako R, et al. Patient-level and system-level determinants of stroke fatality across 16 large hospitals in Ghana and Nigeria: a prospective cohort study. Lancet Glob Health. 2023 Apr;11(4):e575–85.

[8] Edzie EKM, Gorleku PN, Dzefi-Tettey K, Idun EA, Amankwa AT, Aidoo E, et al. Incidence rate and age of onset of first stroke from CT scan examinations in Cape Coast metropolis. Heliyon. 2021 Feb;7(2):e06214.

[9] Kumi F, Bugri AA, Adjei S, Duorinaa E, Aidoo M. Quality of acute ischemic stroke care at a tertiary Hospital in Ghana. BMC Neurol. 2022 Jan 17;22:28.

[10] Sanuade OA, Dodoo FNA, Koram K, de-Graft Aikins A. Prevalence and correlates of stroke among older adults in Ghana: Evidence from the Study on Global AGEing and adult health (SAGE). Busija L, editor. PLOS ONE. 2019 Mar 13;14(3):e0212623.

[11] Tawiah A. Stroke cases rising among young people. 2023; Available from: https://www.graphic.com.gh/news/general-news/stroke-cases-rising-among-young-people.html.

[12] Sarfo FS, Akassi J, Ofori E, Ovbiagele B. Long-term determinants of death after stroke in Ghana: Analysis by stroke types & subtypes. J Stroke Cerebrovasc Dis. 2022 Sep;31(9):106639.

[13] Baatiema L. The knowledge-practice gap: Evidence-based practice for acute stroke care in Ghana. 2018;

[14] Baatiema L, de- Graft Aikins A, Sarfo FS, Abimbola S, Ganle JK, Somerset S. Improving the quality of care for people who had a stroke in a low- /middle-income country: A qualitative analysis of health- care professionals' perspectives. Health Expect. 2020 Apr;23(2):450–60.

[15] Itanyi UD, Nnodu OE. Prevention of stroke and cognitive decline in pediatric population in resource-limited settings. Front Stroke. 2024 Nov 8;3:1390220.

[16] Sahle Adeba T, Mekonen H, Alemu T, Alate T, Melis T. Survival status and predictor of mortality among adult stroke patients in Saint Paul's hospital millennium medical college, Addis Ababa, Ethiopia. SAGE Open Med. 2022 Jul 30;10:20503121221112483.

[17] Kim JS, Lee KB, Roh H, Ahn MY, Hwang HW. Gender Differences in the Functional Recovery after Acute Stroke. J Clin Neurol Seoul Korea. 2010 Dec;6(4):183–8.

[18] Kelly-Hayes M. Influence of Age and Health Behaviors on Stroke Risk: Lessons from Longitudinal Studies. J Am Geriatr Soc. 2010 Oct;58(Suppl 2):S325–8.

[19] Wiredu EK, Nyame PK. Stroke-related mortality at Korle Bu Teaching Hospital, Accra, Ghana. East Afr Med J. 2001 Apr 1;78(4):180–4.

[20] Che B, Shen S, Zhu Z, Wang A, Xu T, Peng Y, et al. Education Level and Long- term Mortality, Recurrent Stroke, and Cardiovascular Events in Patients With Ischemic Stroke. J Am Heart Assoc. 2020 Aug 18;9(16):e016671.

[21] Jackson CA, Sudlow CLM, Mishra GD. Education, sex and risk of stroke: a prospective cohort study in New South Wales, Australia. BMJ Open. 2018 Sep 21;8(9):e024070.

[22] Trimble B, Morgenstern LB. Stroke in Minorities. Neurol Clin. 2008 Nov;26(4):1177–90.

[23] Ariss RW, Minhas AMK, Lang J, Ramanathan PK, Khan SU, Kassi M, et al. Demographic and Regional Trends in Stroke- Related Mortality in Young Adults in the United States, 1999 to 2019. J Am Heart Assoc. 2022 Sep 20;11(18):e025903.

[24] Cruz-Flores S, Rabinstein A, Biller J, Elkind MSV, Griffith P, Gorelick PB, et al. Racial-Ethnic Disparities in Stroke Care: The American Experience: A Statement for Healthcare Professionals From the American Heart Association/American Stroke Association. Stroke. 2011 Jul;42(7):2091–116.

[25] Jenkins C, Ovbiagele B, Arulogun O, Singh A, Calys-Tagoe B, Akinyemi R, et al. Knowledge, attitudes and practices related to stroke in Ghana and Nigeria: A SIREN call to action. PLoS ONE. 2018 Nov 16;13(11):e0206548.

[26] Admas M, Teshome M, Petrucka P, Telayneh AT, Alamirew NM. In-hospital mortality and its predictors among adult stroke patients admitted in Debre Markos Comprehensive Specialized Hospital, Northwest Ethiopia. SAGE Open Med. 2022;

[27] Chang MC, Choo YJ, Seo KC, Yang S. The Relationship Between Dysphagia and Pneumonia in Acute Stroke Patients: A Systematic Review and Meta-Analysis. Front Neurol. 2022 Mar 17;13:834240.

[28] Sadiq AO, Awotidebe AW, Saeys W, Truijen S, Wong TWL, Ng SSM, et al. Prevalence, associated factors and predictors of post stroke pneumonia in a Nigerian population: A retrospective study. 2023;

[29] Akpalu J, Yawson AE, Osei-Poku F, Atiase Y, Yorke E, Adjei P, et al. Stroke Outcome and Determinants among Patients with and without Diabetes in a Tertiary Hospital in Ghana. Stroke Res Treat. 2018 Sep 12;2018:7521351.

30] Price HL, Campbell SG. Isolated Lower Limb Weakness Following Hemorrhagic Stroke: A Case Report. Cureus. 2023;15(5):e38798
