# Supplementary material for: In-hospital survival and predictors of mortality among stroke patients at a tertiary hospital in Ghana: A retrospective cohort study
Source: PLoS One. 2026 Jan 7;21(1):e0340447. doi: 10.1371/journal.pone.0340447 (PMC12795378; doi:10.1371/journal.pone.0340447)
Supplement: Attachment — Submitted filename: Reviewer Report.docx [file pone.0340447.s003.docx]

Dear Editor,

I would like to sincerely thank you for inviting me to review the manuscript submitted to PLOS ONE. I appreciate the trust you have placed in me to contribute to the peer review process. It is an honor to support the journal in maintaining the quality and integrity of scientific publishing.

### Overall Evaluation

This study investigates predictors of survival among stroke patients admitted to Tamale Teaching Hospital (Ghana). The topic is highly relevant, as survival data on stroke patients in sub-Saharan Africa are scarce. The manuscript is valuable and based on a relatively large dataset, but several methodological clarifications, improvements in tables, and more cautious interpretation of results are needed before publication.

### Abstract

- The follow-up period (mean survival: 67 days) is not mentioned.

- A protective effect of diabetes is reported as an unexpected finding, but it is presented without sufficient explanation or cautious interpretation.

- Suggestion: The follow-up duration and key study limitations (e.g., retrospective design) should be stated. Unexpected findings should be reported with greater caution and accompanied by interpretation.

### Methods

- The definition of the outcome is unclear. The text indicates that “survival status” was determined at discharge (alive or dead), but the Kaplan–Meier results report a mean survival of 67 days with follow-up up to 50 days. This creates confusion as to whether the authors considered in-hospital survival only or included post-discharge follow-up.

- Suggestion: Authors should explicitly clarify how the outcome was defined (discharge status only or extended follow-up). Without this, it is difficult to compare results with other studies and to interpret the Kaplan–Meier curves.

- Handling of missing data is not described.

- No justification for sample size or study power is provided.

- Suggestion:

• Clarify the approach to handling missing data.

• Acknowledge limitations due to the absence of stroke severity measures (e.g., NIHSS, mRS).

### Results and Tables

- The results are generally clear, but several issues require attention:

• Some associations (e.g., protective effect of diabetes, HR >100 bpm) need further clarification.

• The Kaplan–Meier survival curve should be more fully described (e.g., stratified by stroke type).

• Table 1: For the variable educational level, it should be clarified how many years of formal education each category represents (e.g., Basic, SHS, Tertiary). Abbreviations (such as SHS) should also be explained in the table footnotes or text for the benefit of international readers.

### Conclusion

- The current conclusion largely repeats the results.

- Suggestion: The conclusion should instead emphasize policy and clinical implications, such as the need for stroke units, closer monitoring of high-risk patients, and targeted screening for risk factors.

### References

- The reference list combines local and international sources but includes some outdated ones. In particular, Hatano 1976, which refers to the old WHO definition of stroke, is outdated.

- Suggestion: This reference should be replaced or supplemented with more recent and comprehensive sources, such as the AHA/ASA 2013 statement or more recent systematic reviews on stroke definition.
