# Supplementary material for: In-hospital survival and predictors of mortality among stroke patients at a tertiary hospital in Ghana: A retrospective cohort study
Source: PLoS One. 2026 Jan 7;21(1):e0340447. doi: 10.1371/journal.pone.0340447 (PMC12795378; doi:10.1371/journal.pone.0340447)
Supplement: Attachment — Submitted filename: Response to Reviewers.docx [file pone.0340447.s006.docx]

**Response to Reviewers**

The Academic Editor

PLOS ONE

Dear Mehdi Sharafi, Assistant Professor,

We sincerely thank you for taking the time to review our manuscript entitled **“Predictors of Survival of Stroke Patients in the Tamale Teaching Hospital: A 3-Year Retrospective Review.”** We appreciate your constructive comments and suggestions, which have helped us to strengthen the paper.

Below, we respond point-by-point to each comment. All changes have been incorporated into the revised manuscript (tracked version attached).

**Response to Reviewers**

| Reviewers Comments | Author Responses |
| --- | --- |
| **Reviewer #1** |  |
| The title needs to be rewritten | We revised the title to clarify the outcome and study design. Suggested title: **“In-hospital survival and predictors of mortality among stroke patients at a Tertiary Hospital in Ghana: a 3-year retrospective cohort study.”** |
| Summarise the abstract introduction | We appreciate the reviewer’s suggestion to make the abstract more concise. In response, we have summarised the Introduction section of the abstract to focus on the essential background and study objective. The revised text now reads:  “Stroke is a leading cause of death and disability globally and imposes a growing burden on Ghana’s healthcare system. This study aimed to estimate in-hospital survival rates and identify predictors of mortality among stroke patients admitted to a tertiary hospital in Ghana over a 3-year period.” |
| Your literature review is weak. Please cite examples of similar studies. | We appreciate the reviewer’s feedback. In response, we have strengthened the literature review by including additional studies on stroke survival and its determinants. Specifically, we have now cited:   - Sarfo FS, et al. *Long-Term Outcomes of Stroke in a Ghanaian Outpatient Clinic*. J Stroke Cerebrovasc Dis. 2018. - Sarfo FS, et al. *Key determinants of long-term post-stroke mortality in Ghana*.(**line 91**) |
| Provide logical reasoning for all comparisons (e.g., why your median survival differs from other studies). | We rewrote the comparison paragraph to state explicitly why median survival may differ across studies: This difference is likely explained by contextual factors such as variation in stroke severity and complication rates, health systems, and variation in sample size (**lines 271-273**) |
| **Reviewer #2** |  |
| 1. The follow-up period (mean survival: 67 days) is not mentioned. | Thank you for pointing this out. We have revised the Methods section of the abstract to include the mean follow-up duration (**Line 35**). It now reads: “Kaplan–Meier survival analysis was used to estimate the survival rate of stroke patients (mean follow-up: 67 days).” |
| 2. A protective effect of diabetes is reported as an unexpected finding, but it is presented without sufficient explanation or cautious interpretation. | We appreciate this important comment. In the revised abstract, we have reported the association between diabetes mellitus and mortality more cautiously, noting that the apparent lower risk may be due to differences in case management or residual confounding (**lines 50 – 53**). The Results section now states: “Diabetes mellitus showed an apparently lower risk of mortality (AHR = 0.62, 95% CI: 0.47–0.82); this finding should be interpreted cautiously as it may reflect differences in case management or residual confounding.” (**lines 252-254**) |
| The definition of the outcome is unclear. The text indicates that “survival status” was determined at discharge (alive or dead), but the Kaplan–Meier results report a mean survival of 67 days with follow-up up to 50 days. This creates confusion as to whether the authors considered in-hospital survival only or included post-discharge follow-up. Suggestion: Authors should explicitly clarify how the outcome was defined (discharge status only or extended follow-up). Without this, it is difficult to compare results with other studies and to interpret the Kaplan–Meier curves. | Thank you for this important observation. We have revised the Materials and Methods section to explicitly state that survival status was determined at hospital discharge only; no post-discharge follow-up was included. The time-to-event variable used for Kaplan–Meier analysis was calculated as the number of days from admission to discharge or in-hospital death. This clarification has been added to the “Study Variables” (**lines 129-132**) and “Data Analysis” (**lines 151-152**) subsections. |
| 3. The follow-up duration and key study limitations (e.g., retrospective design) should be stated. Unexpected findings should be reported with greater caution and accompanied by interpretation. | We have incorporated the mean follow-up time in the Methods section, added a sentence on the retrospective design as a limitation, and rephrased the Conclusion to reflect the cautious interpretation of unexpected findings. The Conclusion now reads: “Given the retrospective design and possible unmeasured confounders, the observed lower mortality risk among diabetic patients should be interpreted with caution, and prospective studies are warranted to confirm these associations.” |
| Handling of missing data is not described. | We appreciate this comment. We have now included a statement describing how missing data were handled. Specifically, patients with incomplete or missing key variables were excluded from the analysis, and the number of excluded cases is reported in **Figure 1** |
| No justification for sample size or study power is provided. | We thank the reviewer for this observation. We have added a sentence acknowledging that no a priori sample size or power calculation was performed because the study included all eligible stroke cases within the 3-year period (census sampling). This has been added to the “Patient Identification” subsection (**lines 113-114**). |
| Suggestion: Acknowledge limitations due to the absence of stroke severity measures (e.g., NIHSS, mRS). | We agree with this suggestion. This has been highlighted in the discussion of limitations. |
| Some associations (e.g., protective effect of diabetes, HR >100 bpm) need further clarification. | We thank the reviewer for this observation. We have added text in the Results section noting that the apparent protective effects of diabetes, elevated heart rate (>100 bpm), and higher respiratory rate may reflect differences in case management, stroke subtype, or residual confounding (**lines 250-252**). We caution readers that these findings should be interpreted carefully and may not indicate true protective effects. |
| The Kaplan–Meier survival curve should be more fully described (e.g., stratified by stroke type). | We appreciate this suggestion. We have added a description of the Kaplan–Meier curves stratified by stroke type (ischemic vs. hemorrhagic) in the Results section. A new figure (**Figure 4**) has been included showing separate curves for each type with log-rank p-values reported. |
| Table 1: For the variable educational level, it should be clarified how many years of formal education each category represents (e.g., Basic, SHS, Tertiary). Abbreviations (such as SHS) should also be explained in the table footnotes or text for the benefit of international readers. | Thank you for this important point. We have now clarified in Table 1 footnotes what each educational category represents: Basic (1–9 years of formal education, primary and junior high school), SHS (10–12 years of formal education, senior high school), and Tertiary (≥13 years, post-secondary education). All abbreviations including SHS have been explained in the footnotes. |
| The current conclusion largely repeats the results.  Suggestion: The conclusion should instead emphasize policy and clinical implications, such as the need for stroke units, closer monitoring of high-risk patients, and targeted screening for risk factors. | Thank you. We have rewritten the Conclusion to focus on policy and clinical implications rather than restating results. The revised Conclusion emphasizes the need to strengthen acute stroke care at TTH (including establishing a dedicated stroke unit or protocolized acute stroke pathway), enhanced monitoring and early management of high-risk features (fever, pneumonia, elevated systolic BP), targeted screening and community education, and further prospective research to confirm observed associations and investigate ethnic and sex-based disparities (**lines 365-374**). |
| The reference list combines local and international sources but includes some outdated ones. In particular, Hatano 1976, which refers to the old WHO definition of stroke, is outdated.  Suggestion: This reference should be replaced or supplemented with more recent and comprehensive sources, such as the AHA/ASA 2013 statement or more recent systematic reviews on stroke definition. | We agree and have replaced the Hatano (1976) citation with the AHA/ASA 2013 expert consensus statement, 'An Updated Definition of Stroke for the 21st Century'. (**line 60**) |
