# Supplementary material for: In-hospital survival and predictors of mortality among stroke patients at a tertiary hospital in Ghana: A retrospective cohort study
Source: PLoS One. 2026 Jan 7;21(1):e0340447. doi: 10.1371/journal.pone.0340447 (PMC12795378; doi:10.1371/journal.pone.0340447)
Supplement: Attachment — Submitted filename: Response_to_Reviewers_auresp_3.docx [file pone.0340447.s008.docx]

**Response to Reviewers**

Ghana, 8th December 2025

Editor

PLOS ONE

Subject: Re-submission of the revised manuscript (ID: PONE-D-25-39162R1)

Dear Editor

We sincerely thank the reviewers for their valuable comments and insightful feedback on this manuscript. Your suggestions have been instrumental in improving both the quality and relevance of the work. We believe that the manuscript now represents an even more significant contribution to research on the health and nutrition of women of reproductive age.

Below, we provide point-by-point responses to each comment. The changes made to the manuscript are highlighted with ‘track changes’ in a separate file.

Once again, thank you for devoting your valuable time to reviewing our manuscript.

Sincerely,

Corresponding Author: Sulemana Baba Abdulai

E-mail: [babasulemanaabdulai@gmail.com](mailto:babasulemanaabdulai@gmail.com)

**Response to the Academic Editor**

| First, it is still unclear whether you assessed predictors of survival or predictors of death given the multiple inconsistencies between the manuscript's title and different parts of the manuscript (eg, text of the methods and results sections and titles of tables 4, 5) in this regard. Consider making that information clear from the title of the manuscript to the end of the manuscript. | We have revised the manuscript to consistently focus on predictors of in-hospital mortality. The title, methods, results, and Tables 1 and 2 have been updated accordingly |
| --- | --- |
| Second, you did not specify the study design in the materials and methods section whilst you clearly specified the design and provided a valid reason why this study was a predictive retrospective cohort. You are therefore urged to copy that information from the response letter and paste it in the first sub-section of the Material and Methods section which would be termed "Study design" | We thank the Academic Editor for this important observation. We acknowledge that although the study design was clearly described in our previous response letter, it was not explicitly stated within the Materials and Methods section of the manuscript. We have now addressed this by adding a dedicated subsection titled **“Study design”** as the first subsection of the Materials and Methods section. This subsection clearly states that the study employed a **retrospective cohort design** and provides the rationale for its appropriateness. The relevant text has been incorporated into the manuscript (Page 6, Lines 101–106). |
| Third, in the response letter, you claimed that you could not stratify data by ischaemic and hemorrhagic stroke subtypes. However, you did not mention that information in the limitations statement of the Discussion section. Consider addressing this issue in the manuscript per se. | We have clarified this issue in the Discussion section by explicitly stating that although stroke subtypes were reported at baseline, stratified analyses by ischaemic and haemorrhagic stroke were not performed, and detailed localization data were unavailable (Page 26, Lines 390–395). |
